# Brain network reorganisation and spatial lesion distribution in systemic lupus erythematosus

**DOI:** 10.1177/0961203320979045

**Published:** 2020-12-13

**Authors:** Maria del C Valdés Hernández, Keith Smith, Mark E Bastin, E. Nicole Amft, Stuart H Ralston, Joanna M Wardlaw, Stewart J Wiseman

**Affiliations:** 1 Centre for Clinical Brain Sciences, University of Edinburgh, Edinburgh, UK; 2 UK Dementia Research Institute, University of Edinburgh, Edinburgh, UK; 3 Usher Institute for Population Health Science and Informatics, University of Edinburgh, Edinburgh, UK; 4 Health Data Research UK, London, UK; 5 University Hospitals Birmingham NHS Foundation Trust, Birmingham, UK; 6 Centre for Genomic and Experimental Medicine, University of Edinburgh, Edinburgh, UK

**Keywords:** Connectome, SLE, network analysis

## Abstract

**Objective:**

This work investigates network organisation of brain structural connectivity in systemic lupus erythematosus (SLE) relative to healthy controls and its putative association with lesion distribution and disease indicators.

**Methods:**

White matter hyperintensity (WMH) segmentation and connectomics were performed in 47 patients with SLE and 47 healthy age-matched controls from structural and diffusion MRI data. Network nodes were divided into hierarchical tiers based on numbers of connections. Results were compared between patients and controls to assess for differences in brain network organisation. Voxel-based analyses of the spatial distribution of WMH in relation to network measures and SLE disease indicators were conducted.

**Results:**

Despite inter-individual differences in brain network organization observed across the study sample, the connectome networks of SLE patients had larger proportion of connections in the peripheral nodes. SLE patients had statistically larger numbers of links in their networks with generally larger fractional anisotropy weights (i.e. a measure of white matter integrity) and less tendency to aggregate than those of healthy controls. The voxels exhibiting connectomic differences were coincident with WMH clusters, particularly the left hemisphere’s intersection between the anterior limb of the internal and external capsules. Moreover, these voxels also associated more strongly with disease indicators.

**Conclusion:**

Our results indicate network differences reflective of compensatory reorganization of the neural circuits, reflecting adaptive or extended neuroplasticity in SLE.

## Key messages

- Brain network organisation in SLE patients and healthy age-matched controls significantly differ in some regions.
- SLE showed greater complexity of connectivity patterns in hub regions.
- SLE patients had more network links with larger fractional anisotropy weights than healthy controls.
- Network differences relate to clusters where brain lesions are more strongly associated with disease indicators.

## Introduction

The human autoimmune disease systemic lupus erythematosus (SLE) affects multiple organ systems, including the brain. Patients commonly experience fatigue,^1^ overt cognitive symptoms and impaired endothelial function,^2^ while the risk of stroke is higher than that of the general population.^3,4^ Cerebral small vessel disease (SVD) has been reported in SLE, but was not associated with SLE disease activity, disease duration nor blood markers of inflammation and endothelial function in a prior analysis of 47 patients that investigated SVD across global brain networks.^5^ The main signature of SVD is the presence of white matter hyperintensities (WMH) on structural brain MRI scans. WMH can be commonly found in the brains of older adults with or without clinical symptoms.^6^ They are negatively associated with cognition,^7^ and increased risk of stroke and dementia,^8^ and are also seen in SLE.^5^ Whether their distribution relates to disease indicators and to specific neuroanatomical signatures different from normal healthy individuals is not known.

Connectomics^9,10^ uses graph theory^11^ to analyse the structural (white matter) and functional (correlated brain activity) brain networks derived from MRI techniques. Previous work that used connectomics in SLE found global network measures related to cognitive abilities and clinical systemic damage, but not active disease.^12^ Another study found that brain connectivity networks in SLE patients had decreased efficiency and increased characteristic path length compared to healthy controls.^13^ Complementary to the previously mentioned study,^12^ regional network degree and nodal efficiency in frontal, occipital and cingulum regions negatively correlated with disease activity in this smaller SLE cohort.^13^ Another study also found abnormal global efficiency and network path length in SLE patients compared to controls despite similar functional hub connectivity measurements.^14^

A new paradigm for understanding the complex network topology of the brain has recently been proposed in which hierarchically equivalent nodes have variable connectivity patterns.^15^ The fact that the brain’s numerous regions with different functional specialisations necessitate a wide variety of connectivity patterns in the supporting structure constitutes the basis of this paradigm. Once global connectivity patterns are assessed, it explores the different degree strengths and hubs in the network. Smith et al.^15^ demonstrated that in healthy adults, dividing the connectome into four tiers based on connectivity degree, the most complex nodes were found in the middle two tiers. This suggested that hierarchical complexity of the human adult connectome is not driven by hub nodes, but rather by nodes mainly in heteromodal integrative regions and to a lesser but still significant extent in more basic sensorimotor and visual-semantic areas.

Since SLE pathophysiology is not related to abnormal brain architecture, hierarchically complex connectivity patterns similar to those present in healthy individuals should exist in SLE. However, to the best of our knowledge there has not been a study on the hierarchical structure of the connectome in SLE patients. Moreover, it is not known if they differ from those of disease-free individuals as a destructive by-product of the disease or are ‘reorganised’ as a compensatory mechanism; for example, to re-route signals between regions to circumvent strategically located WMH. Diffusion tensor magnetic resonance imaging (DT-MRI) tractography studies have shown that, in SLE patients, mean diffusivity —a biomarker of brain white matter integrity— is significantly higher than in age-matched controls^16^ and specifically altered in the corpus callosum, uncinate tracts, thalami and cingula.^17–20^ These findings suggest possible diffuse whole-brain damage represented by the prevalent presence of WMH in these regions. Moreover, another DT-MRI study showed that white matter microstructure in SLE patients is related to disease duration and fatigue.^21^ To progress our understanding of how SLE affects the brain, it is useful to investigate the organisation of brain network connections in relation to underlying disease characteristics, and how the specific spatial location of WMH might also map to hierarchical tiers and function. Such knowledge could lead to useful connectomic biomarkers of SLE disease pathophysiology.

Here, we seek to 1) understand if brain network organisation in SLE differs from controls and if so, 2) to explore whether the differences between networks in SLE patients relate to lesion load and 3) investigate whether the lesion load distribution in SLE patients is related to SLE disease markers.

## Methods

### Subjects

We retrospectively analysed data from a study on SLE and age-matched healthy adults recruited by advertisement from staff working at the University of Edinburgh, the Western General Hospital and the Royal Infirmary, Edinburgh, United Kingdom. SLE patients were examined by a consultant rheumatologist at a specialist SLE clinic between April and December 2014. From the 51 SLE patients who participated in the primary study that provided data for the present study,^5^ we analysed data from the 47 patients that had available connectome data. All patients met the updated American College of Rheumatology 1997 criteria for SLE.^22^ The South East Scotland Research Ethics Committee gave study approval (01, 14/SS/0003). The healthy adults from our control group were recruited to participate in a study approved by the Lothian Research Ethics Committee (REC 05/S1104/45). All participants gave written consent.

### Disease indicators

Current SLE disease activity was assessed using the Systemic Lupus Erythematosus Disease Activity Index 2000.^23^ Increasing level of anti-double-stranded DNA from blood samples was also considered an indicator of disease activity. Accumulated permanent damage from SLE was assessed with the Systemic Lupus International Collaborating Clinics (SLICC)^24,25^ damage index and disease duration. Indicators of endothelial function extracted from SLE patients’ blood samples included von Willebrand Factor (VWF) antigen and homocysteine. We also used the following vascular risk factors: presence vs. absence of hypertension and smoking status from the patients’ medical history, and measures of total cholesterol, homocysteine and anticardiolipin IgG and IgM obtained from the analyses of the blood samples.^5^ Fatigue was assessed using the Fatigue Severity Scale, and fibrinolysis was assessed through D-dimer presence in blood.

### MRI acquisition

All MRI data were acquired using a GE Signa Horizon HDxt 1.5 T scanner (General Electric, Milwaukee, WI, USA) using a self-shielding gradient set with maximum gradient strength of 33 mT m^−1^ and an 8-channel phased-array head coil. The scan protocols included axial T2-, gradient-recalled echo-, fluid-attenuated inversion recovery-, sagittal T2- and high-resolution coronal 3 D T1-weighted volume sequences, and a whole brain DT-MRI acquisition. The DT-MRI protocol from both studies consisted of three T2-weighted and 32 diffusion-weighted (*b* = 1000 s mm^−2^) axial single-shot spin-echo echo-planar (EP) imaging volumes (field of view 240 × 240 mm, matrix 128 × 128, TR 13.75 s, TE 78.4 ms).^5^ Scanning protocol parameters are detailed in Supplementary Table 1 of the Supplementary Material.

### Image processing

Each 3 D T1-weighted volume was parcellated into 85 regions-of-interest (ROI), consisting of 68 cortical (34 per hemisphere) and 16 sub-cortical (eight per hemisphere) regions, plus the brainstem, using the Desikan-Killiany atlas in FreeSurfer (http://surfer.nmr.mgh.harvard.edu).^26^ The results were used to construct the tissue and region-of-interest (ROI) masks for network construction and to constrain the tractography output. WMH and intracranial volume (ICV) were extracted semi-automatically using the MCMxxxVI Lesion Extraction tool (www.sourceforge.net/projects/bric1936), followed by a thorough manual boundary rectification if/where needed, as described in Valdes-Hernandez et al.^27^ Diffusion data were processed using the FMRIB Diffusion Toolbox (FDT) package in FSL.^28^ From it, the BedpostX/ProbTrackX algorithm^28^ was used to perform whole-brain probabilistic tractography. The mean fractional anisotropy (FA) values obtained were used to construct the brain networks. As network nodes were not found at the left and right ventral diencephalon (i.e. hypothalamus), network construction was based on 83/85 ROIs. Details on these procedures and on network construction^29,30^ can be found in the Supplementary Material.

### Network analysis

Two levels of analysis (firstly global then hierarchical tier-based) were implemented to understand characteristics of the connectomes and possible relationships between the two.

We chose three global network metrics based on their suitability and known relevance to the human structural connectome. The number of links relative to the number of nodes in the network is a widely studied property^31^ which can be characterised by the normalised network density and average degree. Globally we compute network density for the unthresholded networks, since network density is fixed by the threshold and so would not differ from participant to participant. The global clustering coefficient assesses the tendency of neighbouring nodes to connect to the same other neighbours. This concept of homophily has been shown to be a particularly evident trait of structural connectomes.^32^ We also computed the hierarchical complexity, to measure the extent of topological/functional diversity across the degree hierarchy of the connectomes.^15,33^

Based on the findings of consistent hierarchical tiers across structural connectomes, we also conducted within-tier network analyses. Following Smith *et al*.,^15^ each structural connectome was split into four tiers based on quartiles of the maximum degree. Tier 1 consisted of all nodes with degree greater than 75% of the maximum degree, Tier 2 of all nodes with degree greater than 50% and up to 75% of the maximum degree, and so on.

We used three metrics to assess tier network topology to correspond to those chosen for global analysis. Thus, in each study participant, the average degree of nodes in each tier was computed to track any consistent differences in the number of links established. Unlike for the global measure of density, this is free to vary within tiers in the thresholded networks. The average local clustering coefficient was computed to assess average levels of homophily in the tiers. Finally, hierarchical complexity was computed within each tier to assess the level of within-tier topological diversity.

### Network-lesion spatial distribution analysis

To investigate the spatial relationship between network topology and lesions in relation to disease indicators in SLE patients, we conducted two analyses.

The first analysis consisted of comparing the lesion distribution in the patients’ network tiers with the lesion distribution in the regions that correspond to the control group’s network tiers, mapped to each SLE patient’s brain (see Supplementary Figure 1). For this, we mapped the tiers in each control subject into an age-relevant (55 years old, as this reflects the age of our cohort) template^34^ (https://datashare.is.ed.ac.uk/handle/10283/1957), hereafter called the ‘study template’, using non-linear registration, via NiftyReg^35^ (http://sourceforge.net/projects/niftyreg/) through TractoR (http://www.tractor-mri.org.uk/diffusion-processing). As a result of this process we obtained a probability distribution map of each tier in the control group which we named “control tiers”. Next, using the same software, we applied non-linear registration to map the “control tiers” to the native space of each SLE patient’s brain and calculated, for each patient, the percentage of WMH and “normal-appearing” grey matter in each mapped tier.

The second analysis consisted of investigating the brain locations where the presence of WMH could be associated with disease indicators and network topology in SLE patients. For this, we co-registered all patients’ structural brain images to the study template using 12-degrees affine registration (as per Dickie *et al*.^34^ and Valdés Hernández *et al*.,^36^ specifically for the case of inter-subject co-alignment of periventricular and deep brain lesions), using the same software tools mentioned above, and applied the space transformation to the WMH binary masks. Then, we generated a) spatial probability maps of WMH for each patient subgroup (e.g. hypertensive/normotensive patients, patients with high/low cholesterol, etc.) and b) a 4D volume of all WMH maps concatenated. Patient subgroups were determined by dichotomising and separating into quartiles the disease indicators listed in the subsection “Disease indicators” above. The threshold used to dichotomise the continuous variables was the median value in the SLE sample.

### Repeatability analysis

We evaluated whether the pattern of similarities/dissimilarities between tiers’ structure of SLE patients and controls could be replicated if the control group included more subjects, and if the “control tiers” were generated using a different criterion. This analysis and its results are explained in the Supplementary Material.

### Statistical analysis

We performed three analyses, using MATLAB 2017 b and SPSS Statistics 21. The first analysis aimed to determine whether the connectome networks differed between SLE and control groups. The second analysis consisted in exploring whether differences between controls’ and patients’ brain networks were spatially related to the lesion load distribution in the SLE group. The third analysis consisted in exploring where the lesion load distribution in SLE patients was related to SLE disease markers and whether this voxel-wise association could be explained by the connectome network characteristics in this group.

For the first analysis we used the Wilcoxon rank sum tests on network metrics between SLE patients and healthy controls. The Benjamini-Hochberg false-detection rate procedure^37^ was implemented afterwards across the entire set of resulting *p*-values with the strict criteria of *q* = 0.05. For each tier, we also calculated the Pearson’s correlation coefficients between groups of the number of times individual ROIs were designated to that tier.

For the second analysis we compared the percentage of WMH volume in the regions that corresponded to each “control” tier in the patient native space with the percentage of WMH volume in the corresponding patient tier using the Wilcoxon matched-pair signed-rank test. Of note, for these comparisons the WMH volume in each tier was adjusted by the tier volume. We also calculated the bootstrapped Pearson’s correlation coefficient between these values using *n* = 1000 samples. Similarly, we evaluated volumetric differences between the “normal-appearing” tissue in these regions by comparing the percentage of “normal-appearing” grey matter volume in each “control” tier in the patient’s image space with the percentage of “normal-appearing” grey matter volume in the patient’s own tier.

For the third analysis, we performed voxel-based statistical comparisons of WMH maps using the Wilcoxon’s rank sum test (i.e. to compare two opposite patient groups: e.g. normotensive vs. hypertensive patients, patients with high cholesterol vs. those with low cholesterol, etc.), and the Kruskal-Wallis test (i.e. to compare the WMH maps from more than two groups, e.g. patients falling in each quartile of the vWF antigen). Voxel-wise false discovery rate was used to correct for multiple comparisons. We also implemented a voxel-wise regression model using the 4 D WMH volume constructed as previously explained and a machine-learning approach. This used the MATLAB function “fitrlinear” to fit a regularised Support Vector Machine regression model with a ridge penalty type optimised through a stochastic gradient descent approach for accuracy. This model was selected due to the high-dimensionality and sparsity of the predictor data. In these regression models our predictor was the probability distribution map of WMH in the sample, the covariates were age and biological sex and the outcome was the disease indicator or the network global measure. Also, to reduce sparsity, each of the 3 D WMH arrays (i.e. these 3 D arrays from the 4 D array used in the models) were resized to the 3 D space limited by the bounding box of the intracranial volume of the study brain template. The regularisation term strength was set at 1/47.

### Data availability statement

MRI and associated meta-data from the healthy control sample are available from the Brain Images of Normal Subjects (BRAINS) Imagebank (https://www.brainsimagebank.ac.uk/) (Job *et al*.^38^) Specifically, the primary study that provided these data is labelled as NIH-DTI^39^ in the Study Provenance Information of this database, available from (https://www.brainsimagebank.ac.uk/datasets) (accessed on 21.10.2019). Brain templates, probability distribution maps, brain network connectivity metrics (global and per tiers) from both patients and controls, and software associated to this publication are all freely available from Edinburgh Datashare (https://datashare.is.ed.ac.uk/handle/10283/3515).^40^ The SLE clinical and imaging data are not publicly available as they contain information that could compromise the privacy of research participants.

## Results

### Subjects

Forty-seven SLE patients of mean age 48.5 (SD 13.7) years had connectome data (Table 1). Less than one-fifth (17%) were hypertensive, none had diabetes, 12.7% were current smokers, and one subject had a history of stroke. Four patients were left-handed and none had neuropsychiatric symptoms. The control group had 47 healthy adults of similar age to the patient group.

**Table 1.** Groups characteristics.

	SLE patients	Healthy controls
Demographics		
*N*	47	47
Age, years (±SD)	48.5 ± 13.7	44.6 ± 11.5
Female	43/47 (91.5%)	41/47 (87.2%)
Steroids	17/47 (36%)	Not applicable
Disease activity		
SLEDAI (Q1 to Q3)	2 (0 to 4)	Not applicable
Anti-double-stranded DNA (Q1 to Q3)	14.9 (8.67 to 29.12)	Not applicable
Permanent damage		
Disease duration, months (Q1 to Q3)	49 (24 to 118)	Not applicable
SLICC (Q1 to Q3)	0 (0 to 1)	Not applicable
Endothelial function		
Von Willebrand Factor antigen (Q1 to Q3)	1.52 (1.27 to 1.85)	Not applicable
Homocysteine (Q1 to Q3)	17 (15 to 20)	Not applicable
Vascular risk factors		
Hypertension (Y/N)	8/47 (17%)	Not applicable
Smoking status	27/47 (57.4%) never, 14/47 (29.8%) previous, 6/47 (12.8%) current smokers	Not applicable
Total cholesterol (Q1 to Q3)	4.9 (4.4 to 5.5)	Not applicable
IgG (Q1 to Q3)	2.95 (1.98 to 5.32)	Not applicable
IgM (Q1 to Q3)	1.65 (1.12 to 3.22)	Not applicable
Fatigue		
Fatigue Scale Scores (Q1 to Q3)	5.5 (4.26 to 6.26)	Not applicable
Fibrinolysis		
D-dimer (Q1 to Q3)	112.5 (69.75 to 172.5)	Not applicable
White matter hyperintensities (WMH)		
Volume (ml)	0.83 (0.017 to 26.667)	Negligible
Global network connectivity measures		
Mean weight	0.4153 (0.0156)	0.3961 (0.0185)
Density (%)	40.97 (3.01)	36.77 (3.47)
*At 25% density*		
Clustering coefficient	0.4602 (0.0305)	0.4949 (0.0197)
Normalised degree variance	0.2879 (0.0240)	0.3050 (0.0290)
Hierarchical complexity	0.4281 (0.1615)	0.3934 (0.1598)
Tier-based network connectivity measures		
Tier 1 average degree	46.82 ± 4.20	45.83 ± 2.51
Tier 2 average degree	32.40 ± 2.61	32.11 ± 2.14
Tier 3 average degree	19.68 ± 1.27	19.50 ± 1.47
Tier 4 average degree	6.75 ± 0.80	6.18 ± 0.86
Tier 1 clustering coefficient	0.3984 ± 0.0377	0.4251 ± 0.0253
Tier 2 clustering coefficient	0.4726 ± 0.0420	0.5208 ± 0.0330
Tier 3 clustering coefficient	0.5397 ± 0.0490	0.5920 ± 0.0389
Tier 4 clustering coefficient	0.4202 ± 0.0797	0.4541 ± 0.0804
Tier 1 hierarchical complexity	0.0204 ± 0.0054	0.0172 ± 0.0061
Tier 2 hierarchical complexity	0.0646 ± 0.0251	0.0598 ± 0.0252
Tier 3 hierarchical complexity	0.1964 ± 0.0723	0.1805 ± 0.0534
Tier 4 hierarchical complexity	0.8411 ± 0.3619	0.7916 ± 0.3471

Values are mean, median (Q1 to Q3), or number (%). SLE = systemic lupus erythematosus, SLEDAI = Systemic Lupus Erythematosus Disease Activity Index, SLICC = Systemic Lupus International Collaborating Clinincs.

### First analysis: Comparison between the SLE patient group and control group connectome networks

#### Brain connectivity

Average weights and number of links were both found to have statistically significantly higher values in SLE patients ($p\;=\;9.95\times 10^{-7},$ Cohen’s *d* = 0.9818 and $p\;=\;1.51\times 10^{-7},$ Cohen’s *d* = 1.0880, respectively) than in controls.

#### Global and hierarchical structural network topology

**Figure 1.**
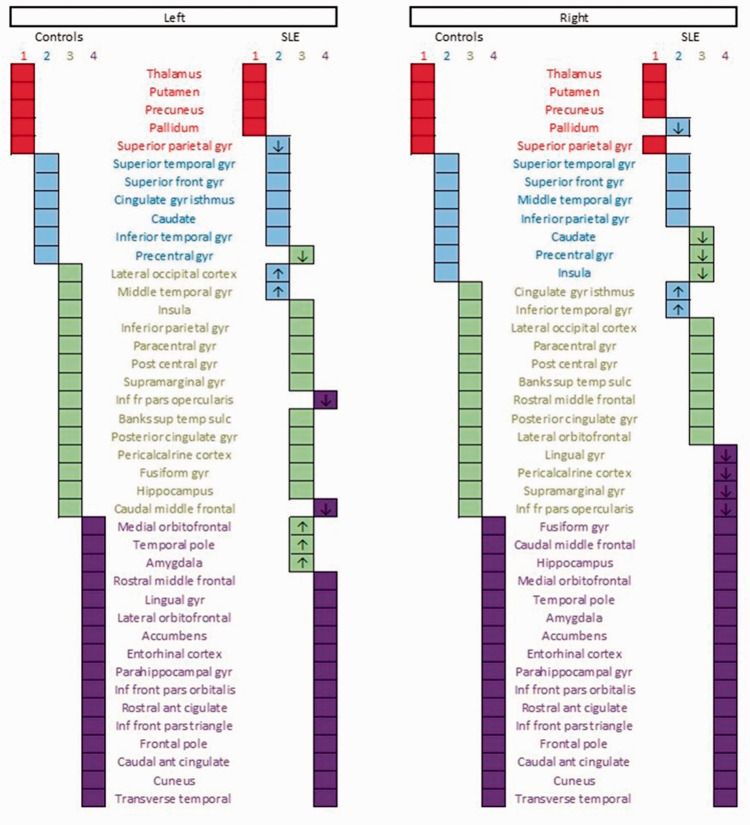
Connectome nodes in controls and SLE patients organized into hierarchical tiers. The expected tier structure is given for left and right regions of interest excluding the brainstem. Diagram confirms large correspondence between controls and SLE patients. However, some nodes in SLE patients are re-organized in the network hierarchy with a tendency for nodes to shift from more important tiers to lower in the tier structure (example: the tier 1 nodes right pallidum and left superior parietal gyrus are placed in tier 2 in SLE, as indicated by the down arrows). Compensatory re-structuring sees some nodes in SLE patients go in the opposite direction, that is, to higher orders in the hierarchy (example: the right inferior temporal gyrus (a tier 3 node) is a tier 2 node in the SLE cohort, as indicated by the up arrow). Four right side tier 3 nodes are noted to “shift down” to lower order tier 4 nodes in SLE. Tier 1 is represented in red, Tier 2 in blue, Tier 3 in green and Tier 4 in purple.

The spatial distribution of the tiers for SLE patients and controls is shown in Figure 1. More details can be found in Supplementary Table 2, complemented by Supplementary Figure 3.

Despite variation in tier distribution in SLE patients and controls (Figure 1, Supplementary Table 2 and Supplementary Figure 4) there was high correlation of the FreeSurfer ROI (i.e. network nodes) placements within tiers between the two groups: *r* = 0.972 for Tier 1, *r* = 0.986 for Tier 2, *r* = 0.813 for Tier 3 and *r* = 0.921 for Tier 4 (Supplementary Figure 3). A few ROIs were inconsistently placed: we observed a tendency for these ROIs of SLE patients to be represented in lower order tiers suggesting a loss of importance in the network hierarchy (see Figure 1 and Supplementary Table 2).

**Figure 2.**
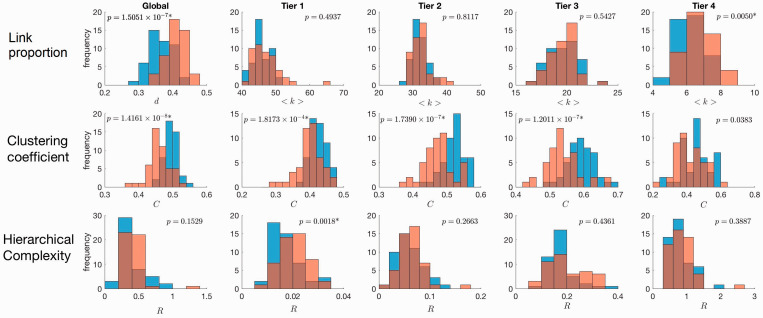
Histograms of network metrics of SLE structural connectomes (orange) and healthy controls (blue). The first left-hand side column shows global metric values and subsequent columns show tier metric values. The first plot in the top row shows the global network density, d, from the unthresholded networks, since global average degree is fixed by the threshold. Average degree, <k>, is shown subsequently in the other columns of the top row for individual tiers. The second row shows global and tier-specific clustering coefficent, C, and the third row shows global and tier-specific hierarchical complexity, R.

The histograms showing the distributions for network density (equivalent to average degree for fixed number of links), clustering coefficient and hierarchical complexity for SLE patients and controls are plotted in the left-hand side column of Figure 2. Analysis within tiers are then plotted in subsequent columns. The *p*-values of the Wilcoxon rank sum tests are shown in the top of each plot with an asterisk (*) indicating where the value passed the false detection rate procedure.

The **network density** was higher in SLE patients (Cohen’s d = 0.6166). The higher average degree in Tier 4 nodes (Cohen’s d = 0.6546) provides an explanation for this. Peripheral SLE nodes have a larger proportion of connections, although there is no sign of a redistribution of this amongst any of the other tiers as the average degree in Tiers 1-3 did not significantly differ between SLE patients and controls.

There was a particularly strong difference in the **clustering coefficient** between the two populations. Clustering was significantly less in SLE than in healthy controls (*Cohen’s d = 1.1235*). Tier based analysis showed this difference across tiers (Figure 1), although the difference did not survive false discovery rate in Tier 4.

Global **hierarchical complexity** was not found to be different between the populations. Individually, however, the complexity of Tier 1 was significantly increased in SLE patients (*Cohen’s d = 0.5281*). Complexity in other tiers did not differ.

### Second analysis: Analysis of the spatial relationship between lesion load and network topology in the SLE group

#### Spatial lesion distribution per tiers

The spatial distribution of the tiers in the SLE patients is detailed in Supplementary Table 2. Given that these refer to network node locations, which are in the cortical and deep grey matter, whereas WMH are mainly in the white matter, the percentage of WMH in the tiers is small compared to the overall WMH volume: median 0.0615% in ICV [0.0323 0.150]. Table 2 shows the WMH load per tier. Tier 1 has the highest percentage of WMH burden across the sample (mean 1.945% of Tier 1 volume, SD 3.332%). Tier 1 mainly covers the thalami, precuneus, left putamen and left globus pallidus. In 30–60% of the patients it also extends to the right putamen and right globus pallidus. The second highest percentage of WMH burden in this sample is in Tier 2 (mean 1.198% of Tier 2 volume, SD 2.054%). This tier is more widely distributed, but in 10–50% of patients it shares the caudate, putamen, globus pallidus and in 45% of patients it includes the brainstem. Tier 3 shares the smallest percentage of WMH burden in the sample (mean 0.718% of Tier 3 volume, SD 1.260%), in the caudate and brainstem. When the control tiers were mapped into the patients’ brain, the distribution of WMH load per tier followed a similar pattern: Tier 1 had most of the WMH followed by Tier 2, and Tier 3 the fewest. However, their share significantly differed (Table 2). The load of WMH in each mapped tier was significantly correlated despite lack of correlation between some of the apparently healthy grey matter (GM) volumes in each mapped tier (Supplementary Table 3).

**Table 2.** SLE patients’ white matter hyperintensity (WMH) load A) by tiers, B) by the regions that correspond to the control tiers and C) by the regions that correspond to the tiers of a wider control group described in Smith et al.^15^ (see repeatability analysis in the Supplementary Material). The mean lesion load in each region is given as percentage of lesion volume in the region (e.g. WMH volume x 100/region volume).

	SLE patients WMH load by tiers (A)	SLE patients WMH load in ROIs corresponding to the control tiers (B)	Related samples Wilcoxon Signed Rank test (between A and B) (p-value)	SLE patients WMH load in ROIs of Smith et al.^13^ repeatability control group (C)	Related samples Wilcoxon Signed rank test (between A and C) (p-value)
Tier 1	1.945 ± 3.332	3.970 ± 4.803	<0.0001	0.318 ± 0.829	<0.0001
Tier 2	1.198 ± 2.054	2.726 ± 4.769	<0.0001	0.253 ± 0.638	<0.0001
Tier 3	0.718 ± 1.260	1.833 ± 3.904	<0.0001	0.222 ± 0.686	<0.0001
Tier 4	0.775 ± 1.958	2.356 ± 4.617	<0.0001	0.820 ± 2.391	0.008
NA region*				0.661 ± 1.304	

Note: *The NA (i.e. not-assigned) region extends through 22/83 of the Desikan-Killiany Atlas ROIs. In these ROIs, tiers were inconsistent for the sample used in Smith et al.,^15^ in less than 1/3 of the subjects. SLE = systemic lupus erythematosus, WMH = white matter hyperintensity.

### Third analysis: Voxel-based associations between white matter hyperintensities, network measures and clinical indicators in the SLE group

#### Spatial lesion distribution in patient groups with differing levels of disease burden

WMH spatial distribution differed in patients with different levels of disease burden, mainly in the periventricular regions, especially in the anterior horns of the lateral ventricles, and in small clusters in the deep white matter. Supplementary Figures 5 to 9 show some examples, but full volume maps in nifti-1 format are available for all variables evaluated (see data availability statement). Patients with more advanced and active disease, endothelial dysfunction (i.e. von Willebrand Factor Antigen), fatigue and presence/high values of vascular risk factors had more WMH towards the deep white matter and optical radiation, compared to those in early disease stages and with absence/low values of vascular risk factors who had the WMH distributed similar to a large non-dense cloud in the periventricular regions. In general, these differences were statistically significant only in periventricular clusters. But for vascular risk factors (e.g. total cholesterol, Supplementary Figure 7 and hypertension, Supplementary Figure 8) statistically significant differences were additionally observed in deep white matter regions. After applying false discovery rate, voxel-wise differences between WMH in patients with high vs. low values of the Fatigue Scale Score and long vs. short disease duration disappeared.

#### Voxel-based regression analysis

**Figure 3.**
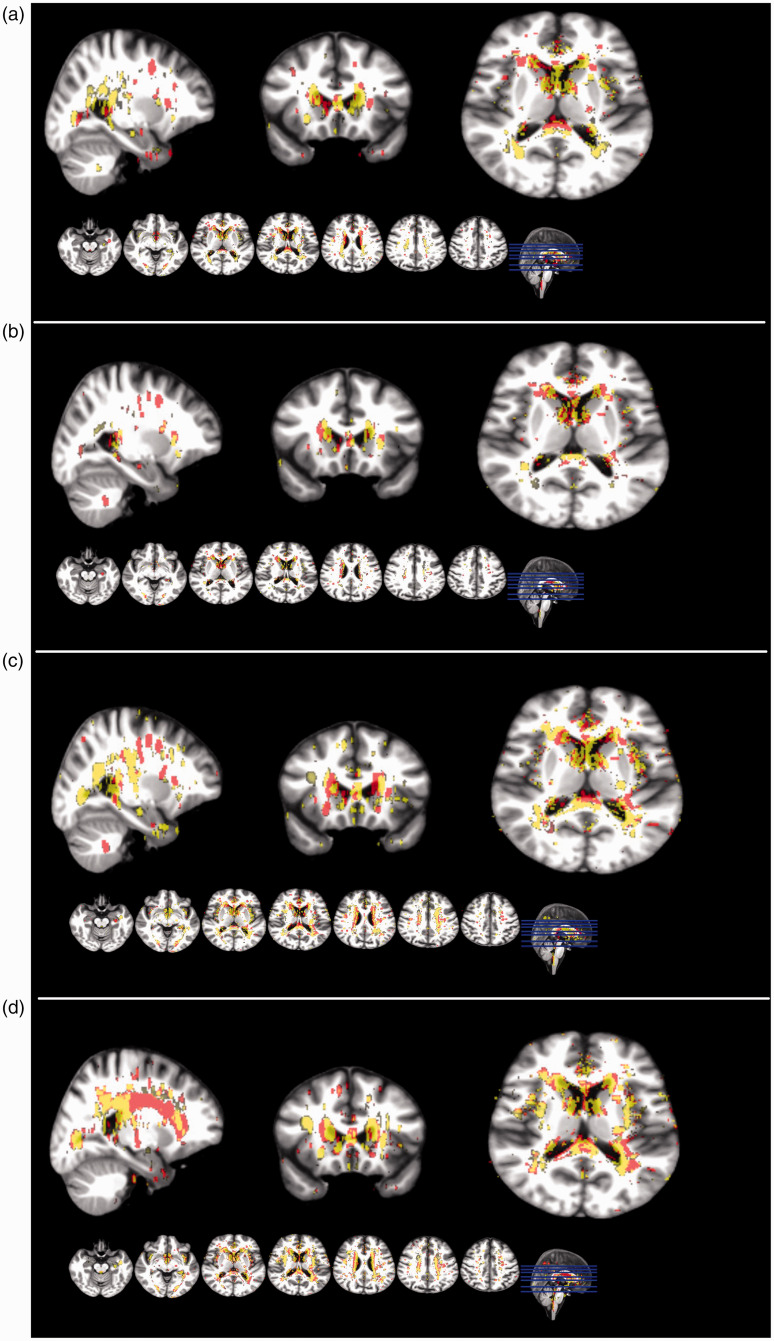
Illustration of the voxel-based association between WMH and: (a) measures of active disease (SLEDAI and anti-double-stranded DNA), (b) endothelial function indicators (von Willebrand factor antigen and homocysteine), (c) measures of clinical disease “damage” (SLICC + Lupus duration), and (d) vascular risk factors (smoker, hypertension (y/n), homocysteine, total cholesterol, anticardiolipin IgG and IgM). From left to right: representative coronal, sagittal and axial slices (above) and five axial slices (below) showing the brain voxels of the study template where positive (red) and negative (yellow) associations were found. The colour intensity is proportional to the strength of the association (normalised to absolute values between 0-maximum B-value for visual representation).

Spatial associations between WMH, all network measures and disease indicators were found after adjusting for age and biological sex. Figure 3 shows representative slices of the study template with the voxels that resulted in positive (green) and negative (red) associations, with the colour intensity being proportional to the strength of the associations. Supplementary Table 4 shows the non-standardised B values of the clusters with the maximum and minimum associations, as well as the median and interquartile range values of these associations in the rest of the voxels where associations were significant. The strongest associations of WMH clusters were observed with clinical parameters extracted from the blood samples (Supplementary Table 4).

**Figure 4.**
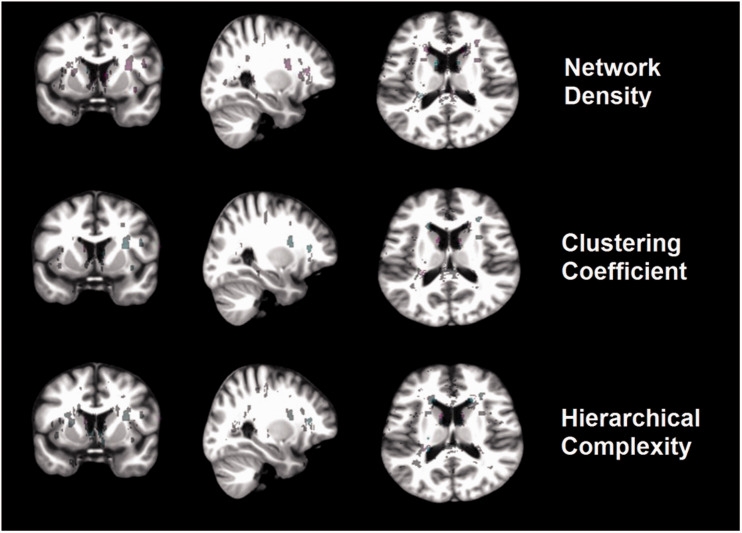
Illustration of the voxel-based associations between WMH and each of the network measures in a representative slice of each plane. The positive (magenta) and negative (cyan) associations are shown mapped on the study template. The colour intensity is proportional to the strength of the association (normalised to values between 0–256 for visual representation).

In all cases, the most relevant negative associations (i.e. in terms of voxel-wise strength and aggregated volume) were found in the periventricular regions of the antero-inferior borders and anterior horns of the lateral ventricles. Although positive associations were found in small clusters scattered throughout the brain, wide clusters located in regions of coalescence between periventricular and deep WMH and in temporo-parietal regions showed stronger associations between WMH and markers of clinical disease severity (SLICC and lupus duration) and vascular risk factors (smoking status, hypertension (y/n), homocysteine, total cholesterol and anticardiolipin IgG and IgM). Interestingly, WMH in the brainstem strongly associated with vascular risk factors, but not with clinical disease manifestations, endothelial dysfunction or measurements of active disease (SLEDAI and anti-double-stranded DNA). WMH in a cluster located in the intersection between the anterior limb of the internal and external capsules in the left hemisphere’s corticopontine white matter tract had stronger negative association with hypertension, total cholesterol, homocysteine and anticardiolipin IgG and IgM and with markers of clinical disease than in the rest of the locations where associations were found. However, when we added the effect of smoking status (i.e. which ranges from 0: non-smoker to 2: current smoker), the association between the total burden of vascular risk factors and WMH in this cluster became positive (e.g. see yellow cluster in the location referred above in Figure 3(d)). In the same cluster, WMH were positively associated with the connectome network density, and negatively associated with the clustering coefficient, and hierarchical complexity (Figure 4). In the mirror region at the right hemisphere, WMH and network parameters were weakly associated in smaller clusters, and the direction of the association was the same as in the left hemisphere.

From all the disease indicators evaluated, D-dimer (Supplementary Table 4 and Supplementary Figure 10) had the strongest association with small WMH clusters, localised in the right hippocampus and right precentral gyrus, in addition to the relevant regions previously mentioned where WMH was more strongly associated with other disease indicators. Smaller clusters in the supramarginal gyrus right, and the pars opercularis and triangularis of the inferior frontal gyrus were also found of relevance. Interestingly, these regions experienced a “shift down” in the number of connections of the network nodes of the SLE patients compared to controls (see Figure 1 and Supplementary Table 2).

## Discussion

Despite inter-individual differences in brain network organization observed across the study sample, the connectome networks of SLE patients and healthy age-matched controls significantly differed in some regions (i.e., pallidum, superior parietal gyri, caudate, precentral gyrus, cingulate, middle and inferior temporal gyri, lateral occipital cortex, temporal pole, amygdala, lingual, supramarginal and medial orbitofrontal gyri, pericalcarine cortex and inferior frontal pars opercularis). SLE patients had statistically larger numbers of links in their networks with generally higher FA weights (i.e., potentially increased white matter tract microstructural integrity) than those of healthy controls. In locations with crossing white matter tracts (i.e., expected low FA values since no preferred diffusion directionality), this result may reflect axonal damage in one of the fiber populations, with consequent stronger diffusion along the remaining white matter fiber bundle, resulting in an apparent increase in FA despite the tissue being not healthy. The voxels exhibiting connectomic differences were coincident with WMH clusters, particularly the left hemisphere’s intersection between the anterior limb of the internal and external capsules. Moreover, these voxels also associated more strongly with disease indicators.

In patients’ brain networks, attempts to bridge ROIs after lesion damage show larger numbers of connections with higher FA (i.e., potentially higher density of coherently ordered myelinated white matter fibers) which are more randomly distributed (i.e., decrease in clustering coefficient) and with greater prevalence in peripheral nodes (i.e., increase in average degree in Tier 4). These re-routings require a greater complexity in the hub node connectivity patterns (i.e. increase in complexity in Tier 1: the tier where the network nodes have most connections) at the expense of a decrease in the nodes’ clustering, precisely due to the higher proportion of lesion load in this region (e.g. native and control-mapped Tier 1 had the highest percentage of lesion load). This decrease in Tier 1’s clustering (i.e., with respect to the healthy controls) may also indicate that either the more redundant connections are dropped or neglected in preference of more variable neighbour-to-neighbour connectivity patterns, favouring the reorganization of hub connections which create more variable connectivity patterns in SLE patients. Smith and colleagues^15^ analysed a larger sample of healthy controls using the same graph-theory-based paradigm and found that Tier 1 nodes were contributing least in the complexity of the connectomes. When the control tiers of such sample were mapped in our patient group, part of the patients’ Tier 1 region was coincident with the region that exhibited great variability amongst this wider control sample, referred to in this study as “NA region”, which exhibited the highest proportion of lesion load. We conjecture that the lack of complexity in healthy Tier 1 hub regions is due to a more ordered core connectivity structure, providing a stable platform to integrate the numerous functionally specialized regions in lower tiers.^41^ The fact that complexity is increased in SLE patients in Tier 1 would indicate that this stable structure is undermined by the disease.

Our results are in-line with those from studies in other diseases. For example, in Parkinson’s disease FA has been found increased in the motor tracts^42^ and selectively decreased in the putamen.^43^ Neural connectivity reorganization after stroke have seen different patterns emerging depending on the time from the stroke event and the extent and location of the stroke,^44^ with specific white matter pathways having greater impact on clinical and functional outcome regardless of the lesion size.^36^ Age, atrophy and inflammation are acknowledged to contribute to network reorganization.^45,46^ However, the pattern of tract-lesion interaction and the influence of white matter disease in this phenomenon are still not very clear. A study in 52 normal individuals at the beginning of the 8th decade of life observed that despite WMH having similar effects on tract infrastructure, whether they be intersecting or nearby, differences in tract water diffusion properties around WMH suggest that tract degeneration may propagate along the white matter tract for intersecting WMH, while in some areas of the brain there is a larger and more localized accumulation of axonal damage in tract tissue nearby a non-connected WMH.^47^ This study also complements findings by other studies in smaller SLE samples, which found altered structural network parameters in SLE patients^13,14^ compared to controls, with only few differences in functional hub measures.^14^

WMH were more strongly associated with disease indicators and with all the global network measures in certain clusters distributed across the longitudinal white matter tracts (i.e. cingulum, arcuate, uncinated and inferior longitudinal fasciculi) and their neighbouring structures. All our regression models, which accounted for age and biological sex, consistently showed associations in these same locations. This may indicate that specific brain locations might be more vulnerable to the presence of WMH and influence brain network topology. From all the disease indicators evaluated, the marker of fibrinolysis had the strongest associations with the same localized clusters of WMH that were associated with the network measures, possibly suggesting a predominant vascular contribution underpinning the brain network differences between patients and controls.

A previous study on global network connectivity measures and cognition in SLE failed to find a relation between structural network connectivity and disease activity^12^ whilst another found a correlation between some regional network properties and disease activity in frontal, occipital and cingulum regions in a sample half the size of the former.^13^ Our voxel-based analyses showed locations where all global network measures and disease indicators, including disease activity, were associated with brain lesions in this patient group, and where connectivity patterns differed from those in a control population. These locations were coincident with those found by another study in SLE had an association between disease activity and resting-state functional connectivity.^48^ Previous results on hierarchical complexity in EEG functional connectivity^30,33^ and in the structural connectome of healthy adults revealed a topological agreement in complexity between structure and function, a paradigm that we have corroborated in SLE.

Our study is the first to conduct a voxel-based network topology analysis of the structural human connectome in SLE patients in relation to disease indicators and lesion distribution and compare the hierarchical complexity of the brain network in SLE with that in normal healthy controls. The comparative analysis of the hierarchical complexity between these two population groups both globally and by structurally different regions in terms of number of connections per-node and connectivity patterns, allowed us to deepen our understanding on the topology and dynamics of these connectivity networks in relation to disease indicators beyond offering descriptive evidence. We, for the first time, use a machine-learning approach to explore the voxel-based associations between brain lesions, disease indicators and network descriptors. Our findings, suggestive of compensatory neuroplasticity in SLE, can inform biological models of neurodegeneration and neuroplasticity in SLE and therapeutic strategies. We used state-of-the-art conventional brain parcellation and tractography methods proven to generate accurate results despite imaging protocol variations. Application of our analysis to larger samples aiming at extending our machine-learning approach for its application in precision medicine is now needed.

## Supplemental Material

sj-pdf-1-lup-10.1177_0961203320979045 - Supplemental material for Brain network reorganisation and spatial lesion distribution in systemic lupus erythematosus Click here for additional data file. Supplemental material, sj-pdf-1-lup-10.1177_0961203320979045 for Brain network reorganisation and spatial lesion distribution in systemic lupus erythematosus by Maria del C Valdés Hernández, Keith Smith, Mark E Bastin, E. Nicole Amft, Stuart H Ralston, Joanna M Wardlaw and Stewart J Wiseman in Lupus
