# Supplementary material for: Brain network reorganisation and spatial lesion distribution in systemic lupus erythematosus
Source: Lupus. 2020 Dec 13;30(2):285–98. doi: 10.1177/0961203320979045 (PMC7854491; doi:10.1177/0961203320979045)
Supplement: sj-pdf-1-lup-10.1177_0961203320979045 - Supplemental material for Brain network reorganisation and spatial lesion distribution in systemic lupus erythematosus [file sj-pdf-1-lup-10.1177_0961203320979045.pdf]

Supplementary Material

Brain network reorganisation and spatial lesion distribution in systemic lupus erythematosus

Authors: Maria del C. Valdés Hernández, Keith Smith, Mark E. Bastin, E. Nicole Amft, Stuart H. Ralston, Joanna M. Wardlaw, Stewart J. Wiseman

Supplementary Methods

Supplementary Table 1 Magnetic resonance imaging scanning parameters

Sequence	Parameter	SLE study	Healthy controls
T1-weighted (T1W)	Acquisition/Orientation	IR/Sag 3D	IR/Cor 3D
	TR/TE/TI (ms)	9.7/4/500	9.7/4/500
	Voxel size (mm^3)	0.94 x 1.3 x 0.94	1 x 1 x 1.3
	Flip angle (^{o})	8	8
	Bandwith (KHz)	15.63	15.63
T2-weighted (T2W)	Acquisition/Orientation	Propeller FSE/Ax 2D	Propeller FSE/Ax 2D
	TR/TE/TI (ms)	9060/100	11320/105
	Voxel size (mm^3)	0.47 x 0.47 x 2.5	1 x 1 x 2
	Flip angle (^{o})		90
	Bandwith (KHz)	25	20.83
FLAIR	Acquisition/Orientation	Ax (2D)	Ax (2D)
	TR/TE/TI (ms)	9402/145/2350	9000/140/2200
	Voxel size (mm^3)	0.47 x 0.47 x 5	1 x 1 x 4
	Bandwith (KHz)	15.63	15.63
SWI/GRE	Acquisition/Orientation	GRE/Ax 2D	GRE/Ax 2D
	TR/TE/TI (ms)	1460/14	900/15
	Voxel size (mm^3)	0.47 x 0.47 x 2.5	1 x 1 x 2
	Bandwith (KHz)	48.8	12.5
DTI	Acquisition/Orientation	Single-shot spin-echo echo-planar/Ax	
	TR/TE/TI (ms)	13750/78.4	
	Voxel size (mm^3)	2 x 2 x 2	
	Directions	72	
	FOV (mm)	240 x 240	
	Matrix	128 x 128	

Ax = axial. Cor = coronal. DTI = diffusion tensor imaging. FOV = field of view. FLAIR = fluit-attenuated inversion recovery. FSE = fast spin echo. GRE = gradient-recalled echo. IR = inversion recovery. ms = milliseconds. Sag = sagittal. TR = time to repitition. SWI = susceptibility-weighted imaging. TE = time to echo.

Structural image processing

Each 3D T1-weighted volume was parcellated into 85 regions-of-interest (ROI), consisting of 68 cortical (34 per hemisphere) and 16 sub-cortical (eight per hemisphere) regions, plus the brainstem, using the Desikan-Killiany atlas in FreeSurfer (http://surfer.nmr.mgh.harvard.edu). The results of the segmentation procedure were then used to construct the tissue and ROI masks for use in network construction and to constrain the tractography output.

The images of the SLE patients underwent additional processing to extract intracranial and WMH volumes. For these, all structural sequences were rigidly linearly aligned to T2-weighted space using the FMRIB's Linear Image Registration Tool (FLIRT) from FSL (http://fsl.fmrib.ox.ac.uk/fsl). The intracranial volume (ICV) was extracted semi-automatically using the T2*-weighted GRE sequence with the Object Extraction Tool in Analyze™ 11.0 followed by manual editing to exclude any erroneously included extracranial tissues. The WMH were extracted semi-automatically using the MCMxxxVI Lesion Extraction tool available from www.sourceforge.net/projects/bric1936. This tool uses co-registered images, which are mapped and fused in the red-green-blue colour space. In this analysis the FLAIR and T2*-weighted images were mapped in the green/red space respectively. After the colour-fusion, a minimum variance quantisation was applied to reduce the bit resolution and facilitate the thresholding selection of each colour band to segment the tissue of interest and allow manual editing of the results for improved accuracy.

Diffusion MRI data processing

Using tools provided by the FMRIB Diffusion Toolbox (FDT) package in FSL, the DT-MRI data were pre-processed to reduce systematic imaging distortions and bulk subject motion artefacts by affine registration of all subsequent EP volumes to the first T2-weighted EP volume (Jenkinson and Smith, 2001). Skull stripping and brain extraction were performed on the registered T2- and diffusion-weighted EP volumes and applied to the fractional anisotropy (FA) volume calculated by DTIFIT in each subject (Behrens et al., 2003). The neuroanatomical ROIs determined by FreeSurfer were then aligned from 3D T1-weighted volume to diffusion space using a cross-modal nonlinear registration method. As a first step, linear registration was used to initialize the alignment of each brain-extracted fractional anisotropy (FA) volume to the corresponding FreeSurfer extracted 3D T1-weighted brain volume using a mutual information cost function and an affine transform with 12 degrees of freedom (Jenkinson and Smith, 2001). Following this initialization, a nonlinear deformation field based method (FNIRT) was used to refine local alignment (Andersson, Jenkinson and Smith, 2007). FreeSurfer segmentations and anatomical labels were then aligned to diffusion space using nearest neighbour interpolation.

Tractography

Whole-brain probabilistic tractography was performed using FSL's BedpostX/ProbTrackX algorithm (Behrens et al., 2007). Probability density functions, which describe the uncertainty in the principal directions of water molecule diffusion, were computed with a two-fibre model per voxel. Streamlines were then constructed by sampling from these distributions during tracking using 100 Markov Chain Monte Carlo iterations with a fixed step size of 0.5 mm between successive points. Tracking was initiated from all white matter voxels and streamlines were constructed in two collinear directions until terminated by the following stopping criteria designed to minimize the amount of anatomically implausible streamlines: (i) exceeding a

curvature threshold of 70 degrees; (ii) entering a voxel with FA below 0.1; (iii) entering an extra-cerebral voxel; (iv) exceeding 200 mm in length; and (v) exceeding a distance ratio metric of 10. The distance ratio metric (Bullitt et al., 2008), excludes implausibly tortuous streamlines.

Network construction

FA-weighted networks were constructed by recording the mean FA value along streamlines connecting all ROI (network node) pairs. The endpoint of a streamline was the first grey matter ROI encountered when tracking from the seed location. Self-connections were removed, and if no streamlines were found between a pair of nodes, the corresponding matrix entry was set to zero. All networks had the same number of nodes, however the number of links varied across participants and cohorts. As many network metrics are dependent on number of nodes and links (Dormann et al., 2009), we binarized all networks to have the same number of links. The minimum link density (proportion of existing links to total possible) of any participant was 29.72%. We rounded this down the nearest 5%, taking the links corresponding to the largest 25% of weights as our binary networks, ensuring maintenance of hierarchical structure (Smith, Abasolo and Escudero, 2017). The left and right ventral diencephalon were not considered as nodes, hence network construction was based on 83 ROIs. These ROIs were grouped into hierarchical tiers depending on their topological characteristics, given by the number of links per node (i.e. node degree). This approach allows us to analyse how similar are the connections established by nodes with the same number of links, in terms of the links of the nodes they are connected to. To characterise the number of links relative to the number of nodes in the network we calculate the normalised network density and the average and maximum degrees. For each brain network, then, we group the nodes in four tiers based on quartiles of the maximum degree. Hence, Tier 1 consisted of all nodes with degree greater than 75% of the maximum degree, Tier 2 of all nodes with degree greater than 50% and up to 75% of the maximum degree, and so on.

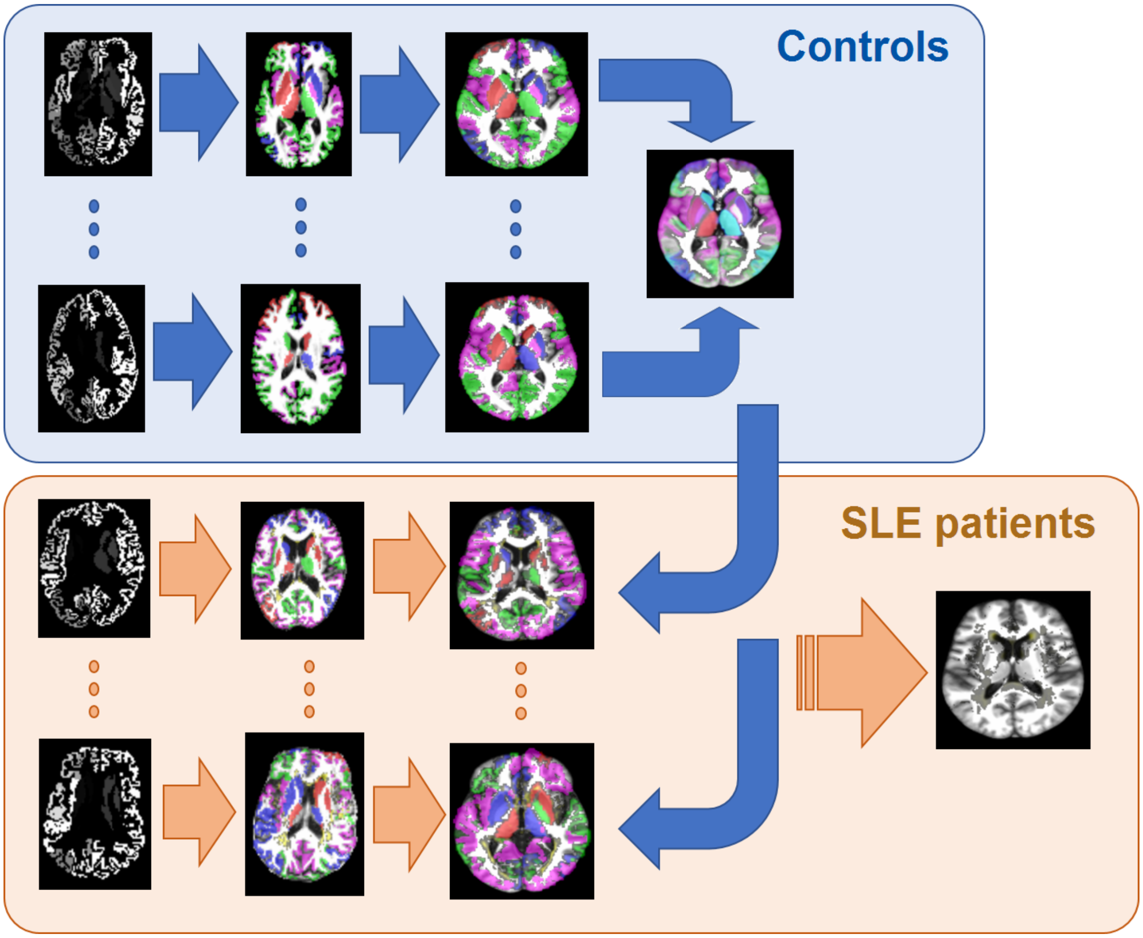

Supplementary Figure 1 Schematic representation of the image processing steps to investigate network topology differences between healthy controls and SLE patients and its relation to lesion distribution. From left to right the 83 regions of interest (ROIs) (column 1) make up the network nodes from each tier (column 2), which are non-linearly mapped in the study template in standard space (column 3) for each of the 47 controls (upper block in blue) and SLE patients (lower block in orange). The control template, where all control tiers are mapped, is mapped into the individual patients' brains (also in standard space). Patients' white matter hyperintensities (in yellow) are also mapped and later combined into a 4D volume for the voxel-based regression analysis.

Repeatability analysis

To evaluate whether the pattern of similarities/dissimilarities between tiers' structure of SLE patients and controls could be replicated if the control group included more subjects and if the "control tiers" were generated using a different criterion, we used the tier atlas of normal subjects generated by Smith et al. (2019).

Each of our "control tiers" constitute a probability distribution map of a named tier in the control group obtained from averaging the tier region from all subjects in the group. Differently, Smith et al. generated a "control tier atlas", where each tier grouped the regions (i.e. FreeSurfer ROIs) that consistently belonged to

the named tier in more than two-thirds of the subjects and grouped the rest of the not-assigned ROIs into a single additional region, which we called “NA region” (see Supplementary Figure 2 below). Of note, this large sample of eighty normal healthy subjects includes the forty-seven subjects that conform the control sample of our study.

We non-linearly registered this atlas to the native space of each patient’s brain using NiftyReg (Modat et al., 2010) (http://sourceforge.net/projects/niftyreg/) through TractoR (http://www.tractor-mri.org.uk/diffusion-processing), and calculated the percentage of white matter hyperintensities (WMH) and grey matter (GM) in each mapped tier (i.e. in each tier of this wider group of healthy controls). Later, we compared these to the percentage of WMH and GM in each of the own patients’ tiers. We also calculated the bootstrapped Pearson’s correlation coefficient between these values using n=1000 samples.

Results of this added analysis are shown in Table 2 of the main manuscript and Supplementary Tables 5 and 6 below.

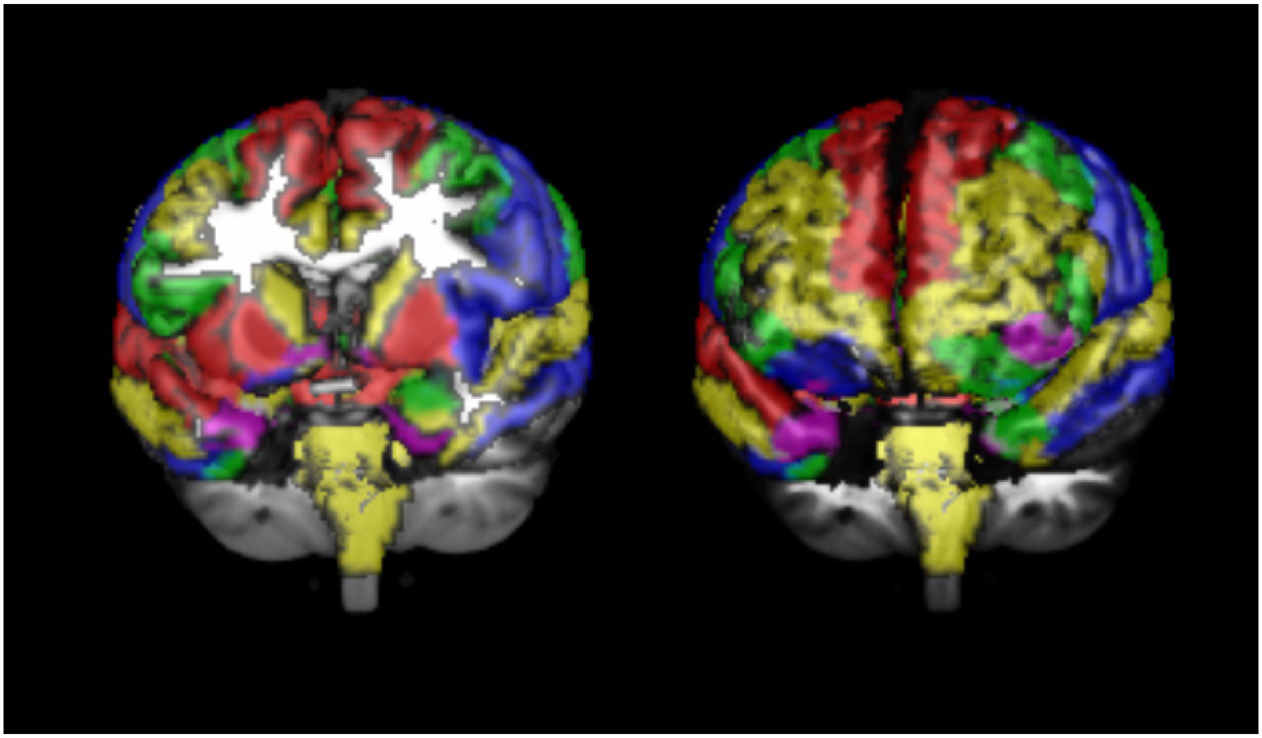

Supplementary Figure 2 3D representation of the control tiers atlas from Smith et al. from 80 normal, healthy volunteers (40 males, 40 females) mapped in our study template. The colours follow the tiers’ representation in Figures 1-3 of the main manuscript: Tier 1 is represented in red, Tier 2 in indigo-blue, Tier 3 in green and Tier 4 in magenta. The 22 brain ROIs that were inconsistent in 1/3 of this control population, which were mapped into a single region called “control NA region”, are represented in yellow. This atlas is used to test repeatability of our results.

Supplementary Results

Supplementary Table 2 Distribution of network tiers in 47 systemic lupus erythematosus (SLE) patients and 47 age- and sex-matched healthy controls

			SLE					Controls					
**ROI no.**	**ROI name (standard FreeSurfer output and ordering)**	Tissue location	**Tier 1**	**Tier 2**	**Tier 3**	**Tier 4**	**Rep Tier**	**Tier 1**	**Tier 2**	**Tier 3**	**Tier 4**	**Rep Tier**	Observations when a ROI/Tier combination differs by > 25% patients between SLE and controls
1	thalamus left	Deep	44	3			1	46	1			1	
2	caudate left	Deep	1	27	19		2	1	32	14		2	
3	putamen left	Deep	36	11			1	42	5			1	
4	pallidum left	Deep	38	9			1	37	10			1	
5	brainstem		1	21	18	7	2		16	23	8	3	
6	hippocampus left	Deep		11	33	3	3		4	23	20	3	
7	* amygdala left	Deep		1	24	22	3			13	34	4	SLE 'shifts up' to Tier 3
8	accumbens left	Deep				47	4				47	4	
10	thalamus right	Deep	46	1			1	47				1	
11	* caudate right	Deep	2	15	30		3		28	18	1	2	SLE 'shifts down' to Tier 3
12	putamen right	Deep	27	20			1	38	9			1	
13	pallidum right	Deep	17	28	2		2	25	22			1	
14	hippocampus right	Deep		1	15	31	4			13	34	4	
15	amygdala right	Deep			3	44	4			3	44	4	
16	accumbens right	Deep				47	4				47	4	
18	banks superior temporal sulcus left	Cortex		3	34	10	3		3	31	13	3	
19	caudal anterior cingulate cortex left	Cortex			9	38	4			8	39	4	
20	***** caudal middle frontal gyrus left***	Cortex			2	45	4			24	23	3	SLE 'shifts down' to Tier 4
21	cuneus left	Cortex			11	36	4			14	33	4	
22	entorhinal cortex left	Cortex			2	45	4				47	4	
23	fusiform gyrus left	Cortex		6	33	8	3			25	22	3	
24	inferior parietal gyrus left	Cortex		17	29	1	3	1	21	22	3	3	
25	inferior temporal gyrus left	Cortex	4	32	11		2		24	22	1	2	
26	cingulate gyrus isthmus left	Cortex	12	31	4		2	11	34	2		2	
27	lateral occipital cortex left	Cortex		25	22		2		22	25		3	
28	lateral orbitofrontal cortex left	Cortex			13	34	4			3	44	4	

29	lingual gyrus left	Cortex		2	16	29	4			21	26	4	
30	medial orbitofrontal gyrus left	Cortex		4	28	15	3			22	25	4	
31	middle temporal gyrus left	Cortex		25	21	1	2	2	20	25		3	
32	parahippocampal gyrus left	Cortex			1	46	4			1	46	4	
33	paracentral gyrus left	Cortex		14	33		3		16	31		3	
34	***** inferior frontal gyrus pars opercularis left***	Cortex		1	23	23	3/4			40	7	3	SLE ‘shifts down’ to Tier 4
35	inferior frontal gyrus pars orbitalis left	Cortex			1	46	4				47	4	
36	inferior frontal gyrus pars triangularis left	Cortex			4	43	4			5	42	4	
37	pericalcarine cortex left	Cortex		1	26	20	3			32	15	3	
38	postcentral gyrus left	Cortex		3	23	21	3		10	32	5	3	
39	posterior cingulate gyrus left	Cortex	1	4	34	8	3		7	39	1	3	
40	***** precentral gyrus left***	Cortex		9	34	4	3		29	18		2	SLE ‘shifts down’ to Tier 3
41	precuneus left	Cortex	33	14			1	41	6			1	
42	rostral anterior cingulate cortex left	Cortex			3	44	4				47	4	
43	rostral middle frontal gyrus left	Cortex			13	34	4			12	35	4	
44	***** superior frontal gyrus left***	Cortex	3	37	7		2	20	25	2		2	SLE ‘shifts down’ to Tier 2
45	* superior parietal gyrus left	Cortex	17	30			2	29	18			1	SLE ‘shifts down’ to Tier 2
46	superior temporal gyrus left	Cortex	7	31	9		2	1	35	11		2	
47	supramarginal gyrus left	Cortex		1	24	22	3		2	25	20	3	
48	* frontal pole left	Cortex			14	33	4			1	46	4	SLE ‘shifts up’ to Tier 3
49	* temporal pole left	Cortex		2	26	19	3			14	33	4	SLE ‘shifts up’ to Tier 3
50	transverse temporal gyrus left	Cortex		1	18	28	4		1	19	27	4	
51	insula left	Cortex		11	28	8	3		2	35	10	3	
52	banks superior temporal sulcus right	Cortex		7	30	10	3		8	35	4	3	
53	caudal anterior cingulate cortex right	Cortex			13	34	4			3	44	4	
54	* caudal middle frontal gyrus right	Cortex			4	43	4			16	31	4	SLE ‘shifts down’ to Tier 4
55	cuneus right	Cortex		1	14	32	4			10	37	4	
56	entorhinal cortex right	Cortex				47	4				47	4	
57	fusiform gyrus right	Cortex			17	30	4			13	34	4	
58	inferior parietal gyrus right	Cortex	3	25	18	1	2	2	32	13		2	
59	inferior temporal gyrus right	Cortex	1	24	21	1	2		20	27		3	
60	cingulate gyrus isthmus right	Cortex	4	23	20		2	1	19	26	1	3	
61	lateral occipital cortex right	Cortex		16	30	1	3		11	36		3	

62	lateral orbitofrontal cortex right	Cortex		2	23	22	3			24	23	3	
63	lingual gyrus right	Cortex			19	28	4			25	22	3	
64	medial orbitofrontal gyrus right	Cortex			3	44	4				47	4	
65	middle temporal gyrus right	Cortex	2	34	11		2	2	33	12		2	
66	parahippocampal gyrus right	Cortex			1	46	4				47	4	
67	paracentral gyrus right	Cortex		7	39	1	3		3	42	2	3	
68	***** inferior frontal gyrus pars opercularis right***	Cortex			10	37	4			37	10	3	SLE 'shifts down' to Tier 4
69	inferior frontal gyrus pars orbitalis right	Cortex			8	39	4			13	34	4	
70	* inferior frontal gyrus pars triangularis right	Cortex			7	40	4			22	25	4	SLE 'shifts down' to Tier 4
71	pericalcarine cortex right	Cortex		1	16	30	4			25	22	3	
72	postcentral gyrus right	Cortex		1	35	11	3		4	40	3	3	
73	posterior cingulate gyrus right	Cortex		4	35	8	3		2	32	13	3	
74	***** precentral gyrus right***	Cortex		4	43		3		26	21		2	SLE 'shifts down' to Tier 3
75	precuneus right	Cortex	30	17			1	36	11			1	
76	rostral anterior cingulate cortex right	Cortex			1	46	4				47	4	
77	rostral middle frontal gyrus right	Cortex		3	34	10	3	1	6	34	6	3	
78	superior frontal gyrus right	Cortex	2	35	10		2	7	39	1		2	
79	superior parietal gyrus right	Cortex	28	19			1	38	9			1	
80	superior temporal gyrus right	Cortex	1	41	5		2	3	40	4		2	
81	***** supramarginal gyrus right***	Cortex		1	19	27	4		1	40	6	3	SLE 'shifts down' to Tier 4
82	frontal pole right	Cortex			7	40	4			2	45	4	
83	temporal pole right	Cortex				47	4				47	4	
84	transverse temporal gyrus right	Cortex			12	35	4		1	16	30	4	
85	insula right	Cortex		23	24		3		27	20		2	
**Nodes with no participants**			**59/83**	**28/83**	**13/83**	**24/83**		**61/83**	**39/83**	**20/83**	**26/83**		
**Nodes with more than 75% of participants**			**4/83**	**3/83**	**4/83**	**22/83**		**8/83**	**3/83**	**9/83**	**20/83**		

*Legend: **ROI no.** refers to the 83 regions of interest (ROIs) making up the network nodes from each tier, as per FreeSurfer labels of the Desikan-Killiany atlas (from 1 to 85, excluding items 9 and 17 being the left and right ventral diencephalon respectively).*
Values are number of participants. For example, the left caudate is categorised as Tier 1 in 44 SLE patients, and so on.
***Rep Tier** (i.e. Representative Tier) indicates tier with highest count.*
*Few ROIs inconsistently belong to different tiers, as per notes in last column: here there is a tendency for SLE to move from higher order tiers to lower order tiers, i.e., loss of connections, highlighted as * where the ROI/Tier combination differs by more than 25% between SLE and controls and ** (and **bold italics**) by more than 35%. As an example: in controls the left caudal middle frontal gyrus has 24 and 23 participants in Tier 3 and Tier 4 respectively, whereas there are 45 SLE patients with this ROI categorised as Tier 4. Conceptually, shifting from Tier 1 to 2, from 2 to 3, and from 3 to 4 indicates loss of connections which is considered to be loss of importance in the network hierarchy. Even*

though there are fewer of them, Tier 1 ROIs are highly connected to the rest of the network relative to Tier 2, Tier 2 to 3, and so on. More nodes in Tier 4 implies greater inequality in the network hierarchy. Three ROI/Tier combinations go in the opposite direction: the left amygdala, left frontal pole and left temporal pole show SLE patients 'shifting up' tiers implying increased connectivity importance of those nodes.

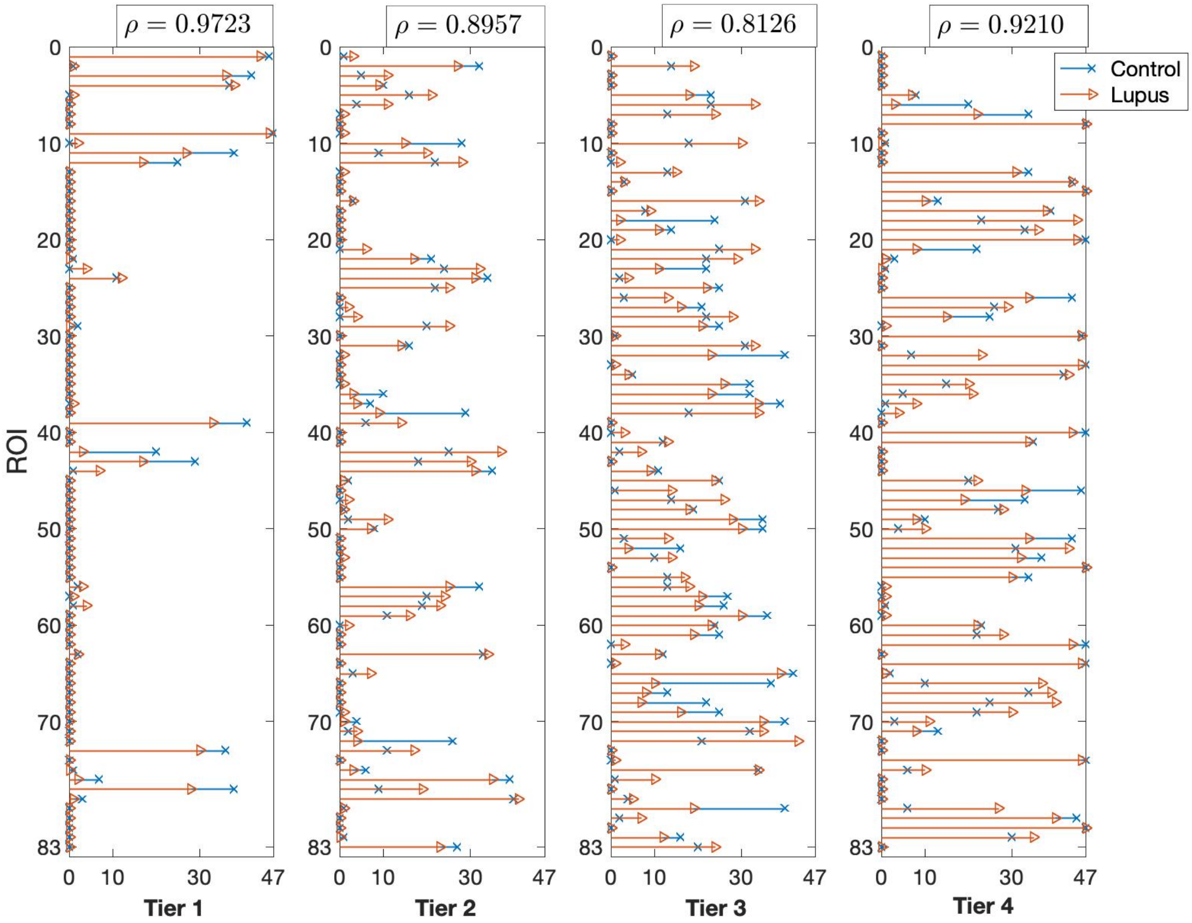

Supplementary Figure 3 Distribution of network tiers in 47 systemic lupus erythematosus (SLE) patients and 47 age- and sex-matched healthy controls. The 83 regions of interest (ROIs) making up the network nodes from each tier are shown along the y-axis following the same order of the FreeSurfer labels of the Desikan-Killiany atlas (from 1 to 85) excluding the ventral diencephalon (left and right). The x-axis represents the number of subjects from each group for each specific tier.

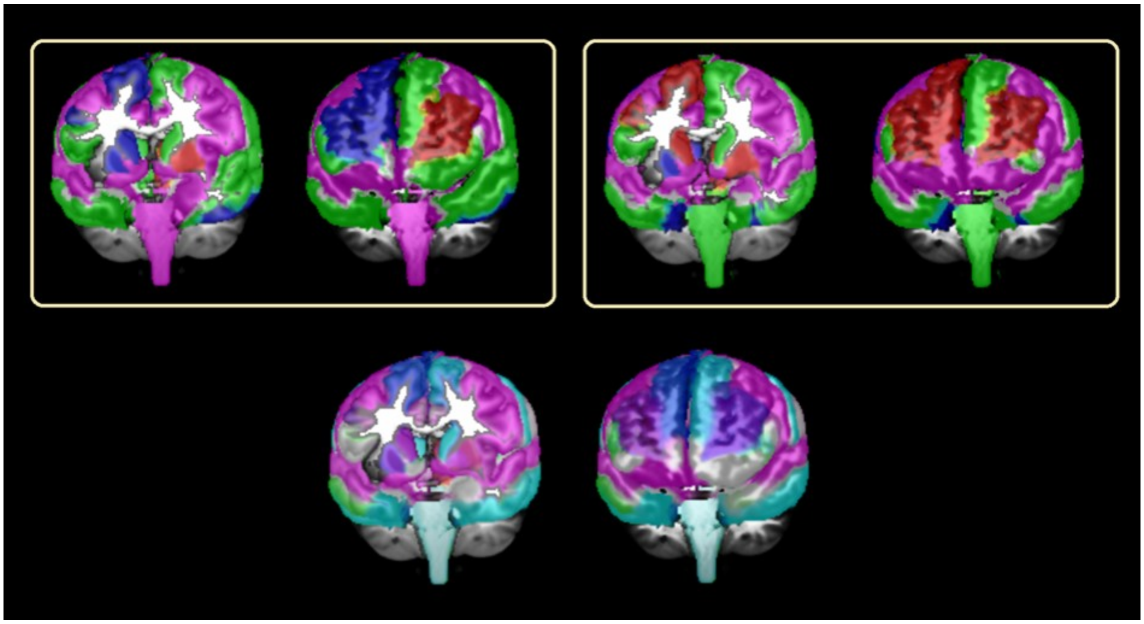

Supplementary Figure 4 Illustration of the tier's variability in control subjects. Enclosed in rectangles, in the upper row, 3D views of the tiers from two subjects mapped in the study template. In the bottom row is a representation of the Control's Tiers Template, which is a probabilistic map of the tiers from all controls. Observe the purple shades generated from combinations of Tiers 1 and 2, cyan regions generated from combinations from Tiers 2 and 3 (mainly), and white-grey-ish areas with the larger variability. Tier 1 is represented in red, Tier 2 in indigo blue, Tier 3 in green and Tier 4 in magenta.

Supplementary Table 3 Pearson's cross-correlations of the percentage of SLE patient's WMH and GM volume in the network own's tiers with the WMH and GM volume in the regions that correspond to the control's tiers. Bootstrap with n=1000 was used to calculate these correlations. The p-values of all correlations involving WMH were less than 0.0001. GM Pearson's r and (p-values) are tabulated.

	SLE's WMH in Tier1	SLE's WMH in Tier 2	SLE's WMH in Tier 3	SLE's WMH in Tier 4	SLE's WMH in Control's Tier 1	SLE's WMH in Control's Tier 2	SLE's WMH in Control's Tier 3	SLE's WMH in Control's Tier 4
SLE's WMH in Tier 1	1	.832**	.707**	.754**	.893**	.795**	.785**	.797**
SLE's WMH in Tier 2		1	.846**	.930**	.925**	.961**	.944**	.926**
SLE's WMH in Tier 3			1	.883**	.928**	.915**	.906**	.946**
SLE's WMH in Tier 4				1	.888**	.971**	.992**	.969**
SLE's WMH in Control's Tier 1					1	.941**	.922**	.944**
SLE's WMH in Control's Tier 2						1	.983**	.978**
SLE's WMH in Control's Tier 3							1	.979**
SLE's WMH in Control's Tier 4								1
	SLE's GM in Tier1	SLE's GM in Tier 2	SLE's GM in Tier 3	SLE's GM in Tier 4	SLE's GM in Control's Tier 1	SLE's GM in Control's Tier 2	SLE's GM in Control's Tier 3	SLE's GM in Control's Tier 4
SLE's GM in Tier 1	1	-0.084 (0.577)	0.037 (0.805)	0.122 (0.413)	0.197 (0.185)	0.015 (0.921)	-0.054 (0.719)	0.054 (0.720)
SLE's GM in Tier 2		1	-.373** (0.010)	-0.107 (0.473)	0.032 (0.828)	0.244 (0.099)	0.218 (0.141)	0.221 (0.135)
SLE's GM in Tier 3			1	-0.060 (0.690)	0.069 (0.647)	.352* (0.015)	.337* (0.021)	.380** (0.008)
SLE's GM in Tier 4				1	.439** (0.002)	.383** (0.008)	.353* (0.015)	.451** (0.001)
SLE's GM in Control's Tier 1					1	.520** (<0.0001)	.481** (0.001)	.589** (<0.0001)
SLE's GM in Control's Tier 2						1	.980** (<0.0001)	.911** (<0.0001)
SLE's GM in Control's Tier 3							1	.903** (<0.0001)
SLE's GM in Control's Tier 4								1

GM = grey matter, SLE = systemic lupus erythematosus, WMH = white matter hyperintensity.

Supplementary Table 4 Numerical results from the voxel-based regression analysis between WMH voxel probabilistic values and the variables tabulated, adjusted age and biological sex.

Group variable	Outcome (dependent) variable	Positive values B median [QR1 QR3]	Negative values B median [QR1 QR3]	Range B
Global network variables	Average Degree	0 [0 0]	0 [0 0]	-0.000016 to 0.000004
	Clustering Coefficient	0 [0 0]	0 [0 0]	-0.000005 to 0.000032
	Hierarchical Complexity	0 [0 0]	0 [0 0]	-0.000015 to 0.000107
Average Degree in each tier	Tier1 Average Degree	0 [0 0]	-0.000001 [-0.000003 0]	-0.00067 to 0.00199
	Tier2 Average Degree	0 [0 0]	-0.000001 [-0.000003 0]	-0.000465 to 0.000811
	Tier3 Average Degree	0 [0 0]	0 [-0.000002 0]	-0.000379 to 0.000932
	Tier4 Average Degree	0 [0 0]	0 [0 0]	-0.00009 to 0.000156
Clustering Coefficient in each tier	Tier1 Clustering Coefficient	0 [0 0]	0 [0 0]	-0.000009 to 0.000008
	Tier2 Clustering Coefficient	0 [0 0]	0 [0 0]	-0.000009 to 0.000017
	Tier3 Clustering Coefficient	0 [0 0]	0 [0 0]	-0.000005 to 0.000098
	Tier4 Clustering Coefficient	0 [0 0]	0 [0 0]	-0.00003 to 0.000064
Hierar-chical Complexity in each tier	Tier1 Hierarchical Complexity	0 [0 0]	0 [0 0]	-0.000006 to 0.000001
	Tier2 Hierarchical Complexity	0 [0 0]	0 [0 0]	-0.000007 to 0.000021
	Tier3 Hierarchical Complexity	0 [0 0]	0 [0 0]	-0.00001 to 0.00003
	Tier4 Hierarchical Complexity	0 [0 0]	0 [0 0]	-0.000051 to 0.00016
Fibrinolysis	D-dimer	0.00001 [0.000003 0.000042]	-0.000043 [-0.000208 -0.000007	-0.027818 to 0.286425
Fatigue	Fatigue Severity Scale	0 [0 0.000001]	0 [-0.000001 0]	-0.002507 to 0.000222
Endothelial dysfunction	F8 von Willebrand Factor Antigen	0 [0 0]	0 [0 0]	-0.000103 to 0.000141
	Homocysteine	0.000001 [0 0.000004]	0 [-0.000001 0]	-0.003144 to 0.001394
Permanent damage	Lupus Duration	0.000057 [0.000011 0.000301]	-0.000007 [-0.000032 -0.000001]	-0.004489 to 0.008022
	SLICC	0 [0 0]	0 [0 0]	-0.000705 to 0.000118
Disease activity	SLEDAI	0 [0 0.000001]	0 [-0.000001 0]	-0.002064 to 0.000356
	Anti-double-stranded DNA	0.00001 [0.000002 0.000053]	-0.000003 [-0.000011 -0.000001]	-0.002935 to 0.003532
Vascular risk factors	Smoker	0 [0 0.000001]	0 [0 0]	-0.000703 to 0.000083
	Anticardiolipin IgM	0.000003 [0.000001 0.000015]	-0.000001 [-0.000002 0]	-0.000457 to 0.002316
	Anticardiolipin IgG	0.000001 [0 0.000009]	0 [-0.000001 0]	-0.000367 to 0.002877
	Hypertension (Y/N)	0 [0 0]	0 [-0.000001 0]	-0.000683 to 0.000042
	Total Cholesterol	0 [0 0]	0 [0 0]	-0.000152 to 0.000736

Supplementary Table 5 Pearson's correlations of the percentage of SLE patient's WMH volume in the network own's tiers and in the regions that correspond to the control's tiers, with the percentage of patient's WMH volume in the tiers of the extended control group used in Smith et al. (2019) (repeatability analysis). Bootstrap with n=1000 was used to calculate these correlations. The p-values of all correlations tabulated were less than 0.0001.

	SLE's WMH in Tier1	SLE's WMH in Tier 2	SLE's WMH in Tier 3	SLE's WMH in Tier 4	SLE's WMH in Control's Tier 1	SLE's WMH in Control's Tier 2	SLE's WMH in Control's Tier 3	SLE's WMH in Control's Tier 4
SLE's WMH in Tier 1 of the extended control sample	.779**	.928**	.910**	.971**	.916**	.968**	.975**	.984**
SLE's WMH in Tier 2 of the extended control sample	.731**	.881**	.907**	.940**	.882**	.919**	.941**	.960**
SLE's WMH in Tier 3 of the extended control sample	.734**	.896**	.859**	.973**	.862**	.932**	.967**	.932**
SLE's WMH in Tier 4 of the extended control sample	.758**	.886**	.877**	.954**	.873**	.910**	.952**	.936**
SLE's WMH in region NA of the extended control sample	.845**	.944**	.878**	.952**	.933**	.946**	.967**	.941**

Supplementary Table 6 Pearson's correlations of the percentage of SLE patient's grey matter (GM) volume in the network own's tiers and in the regions that correspond to the control's tiers, with the percentage of patient's GM volume in the tiers of the extended control group used in Smith et al. (2019) (repeatability analysis). Bootstrap with n=1000 was used to calculate these correlations. Pearson's r and (p-values) are tabulated.

	SLE's GM in Tier1	SLE's GM in Tier 2	SLE's GM in Tier 3	SLE's GM in Tier 4	SLE's GM in Control's Tier 1	SLE's GM in Control's Tier 2	SLE's GM in Control's Tier 3	SLE's GM in Control's Tier 4
SLE's GM in Tier 1 of the extended control sample	0.232 (0.117)	.566** (<0.0001)	0.069 (0.644)	0.222 (0.133)	0.236 (0.110)	.470** (0.001)	.369* (0.011)	.417** (0.004)
SLE's GM in Tier 2 of the extended control sample	0.138 (0.354)	.501** (<0.0001)	0.183 (0.217)	0.240 (0.104)	0.213 (0.151)	.518** (<0.0001)	.409** (0.004)	.513** (<0.0001)
SLE's GM in Tier 3 of the extended control sample	0.115 (0.443)	.451** (0.001)	0.211 (0.154)	0.194 (0.192)	0.146 (0.327)	.496** (<0.0001)	.391** (0.007)	.464** (0.001)
SLE's GM in Tier 4 of the extended control sample	.308* (0.035)	-0.057 (0.702)	.350* (0.016)	0.161 (0.279)	0.127 (0.394)	0.222 (0.133)	0.118 (0.429)	0.255 (0.083)
SLE's GM in Region NA of the extended control sample	0.001 (0.995)	.499** (<0.0001)	0.240 (0.104)	0.187 (0.208)	0.193 (0.193)	.708** (<0.0001)	.631** (<0.0001)	.539** (<0.0001)

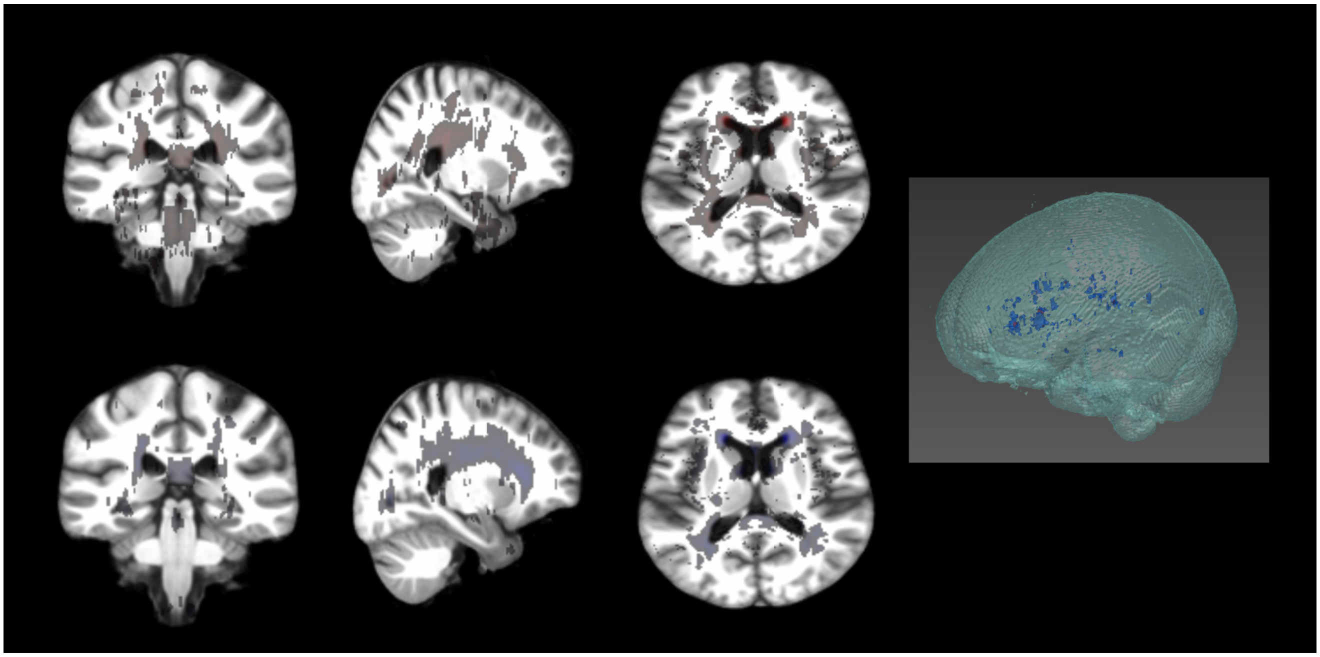

Supplementary Figure 5 Probability distribution map of the WMH in SLE patients with disease duration above the median value of the sample (upper row, red) and below (lower row, blue), and a 3D representation of the voxels where differences between both maps were statistically significant ($p < 0.05$ - blue and $p < 0.01$ - red).

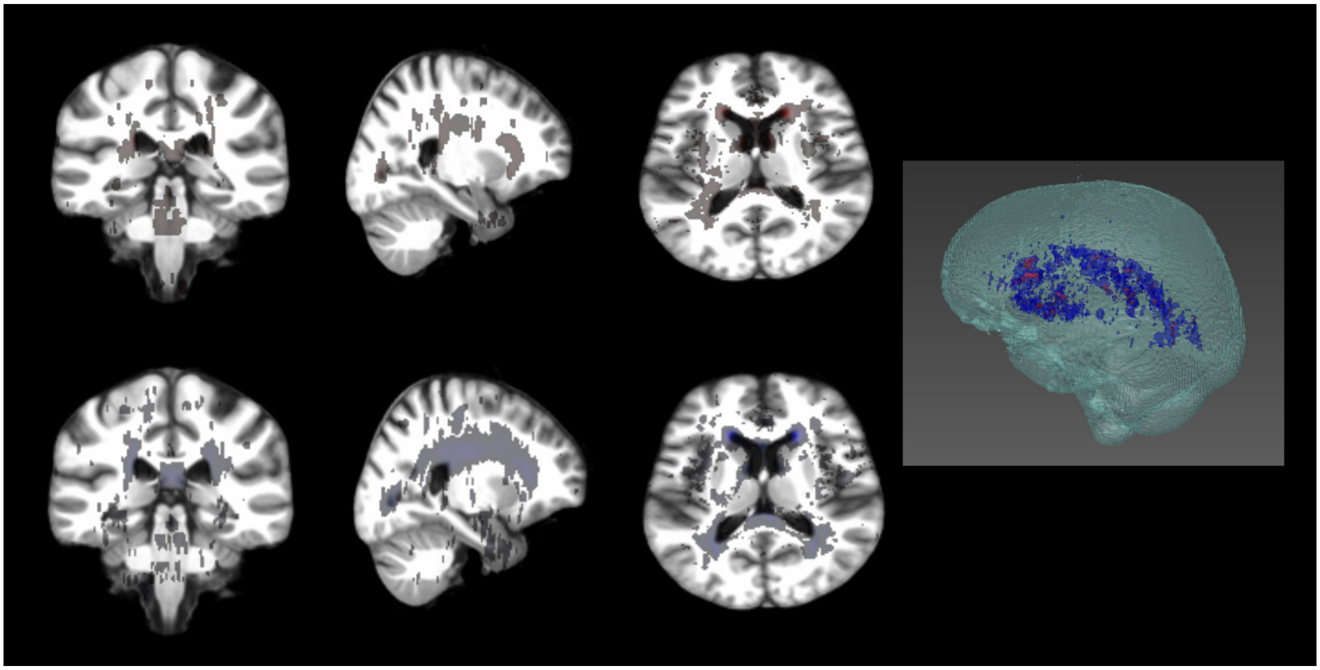

Supplementary Figure 6 Probability distribution map of the WMH in SLE patients with Fatigue Severity Scale values above the median value of the sample (upper row, red) and below (lower row, blue), and a 3D representation of the voxels where differences between both maps were statistically significant ($p < 0.05$ - blue and $p < 0.01$ - red).

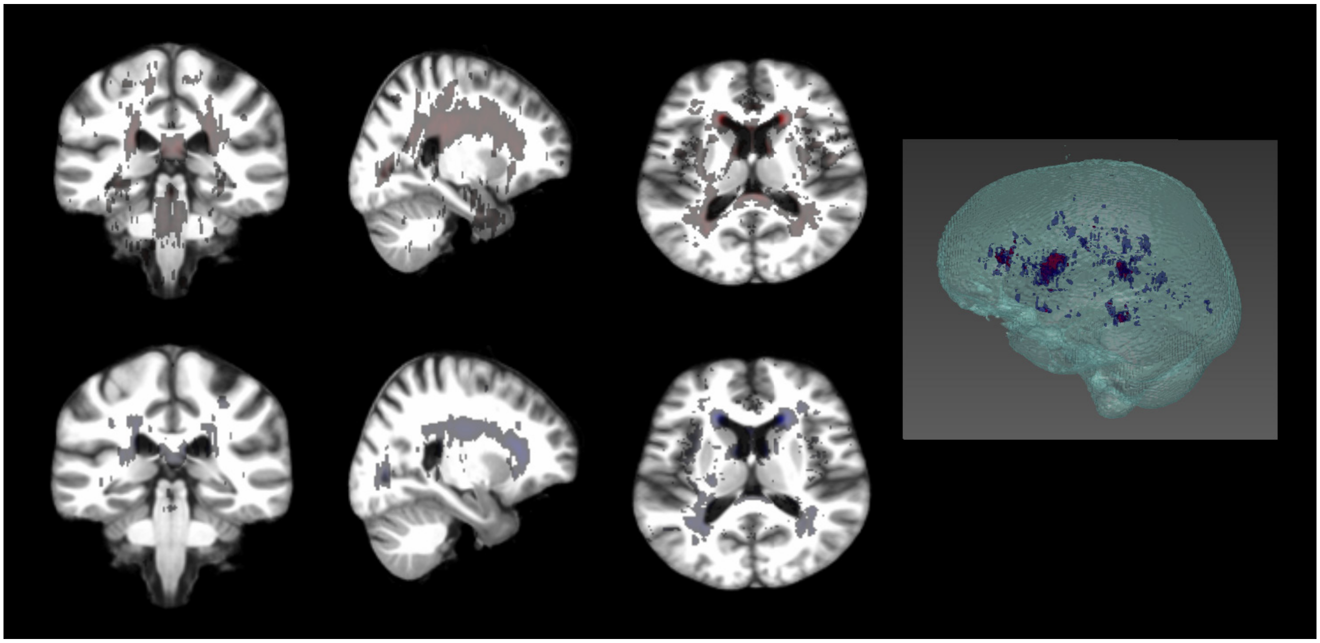

Supplementary Figure 7 Probability distribution map of the WMH in SLE patients with total cholesterol above the median value of the sample (upper row, red) and below (lower row, blue), and a 3D representation of the voxels where differences between both maps were statistically significant ($p < 0.05$ - blue and $p < 0.01$ - red).

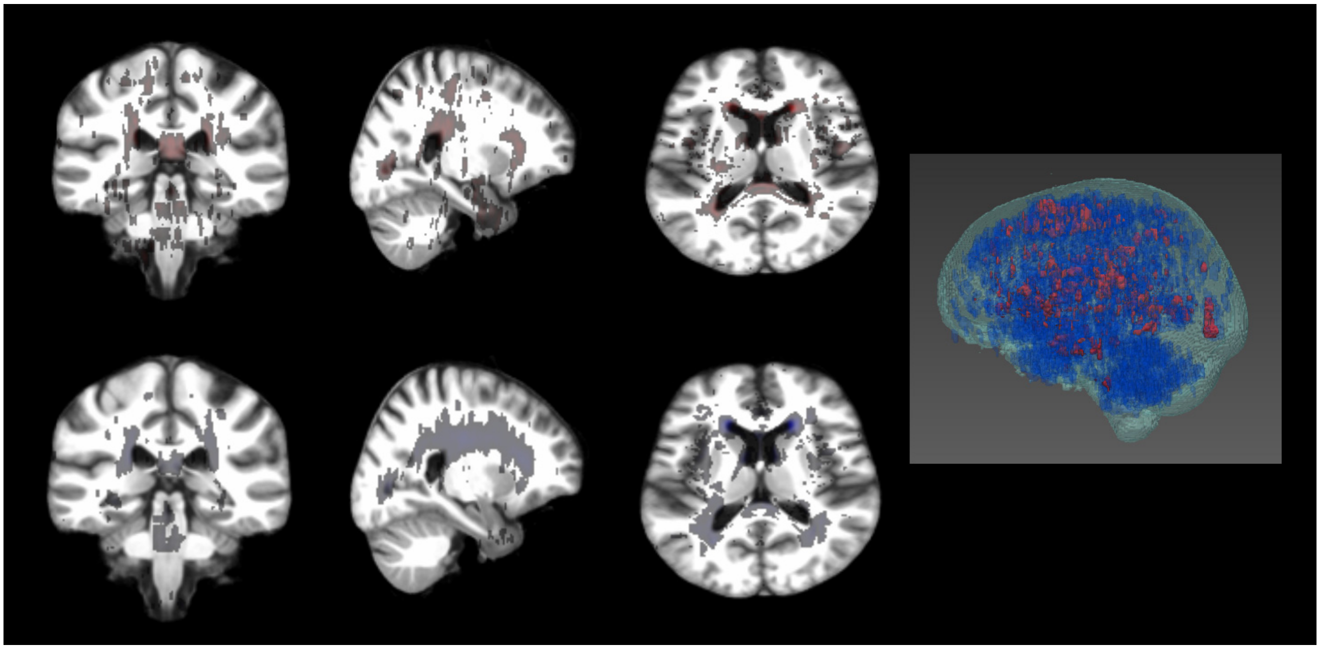

Supplementary Figure 8 Probability distribution map of the WMH in hypertensive (upper row, red) and normotensive (lower row, blue) patients, and a 3D representation of the voxels where differences between both maps were statistically significant ($p < 0.05$ - blue and $p < 0.01$ - red).

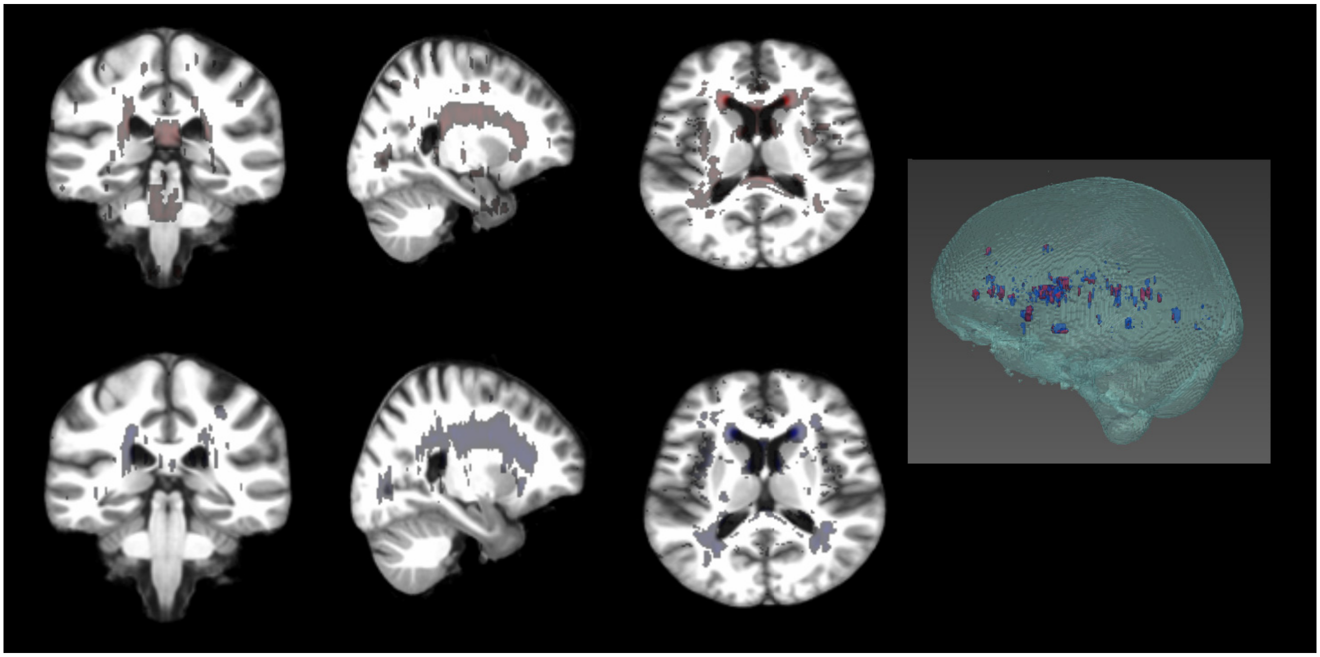

Supplementary Figure 9 Probability distribution map of the WMH in SLE patients with SLEDAI scores in the 4th (upper row, red) and 1st quartiles (lower row, blue), and a 3D representation of the voxels where differences between groups were statistically significant ($p < 0.05$ - blue and $p < 0.01$ - red).

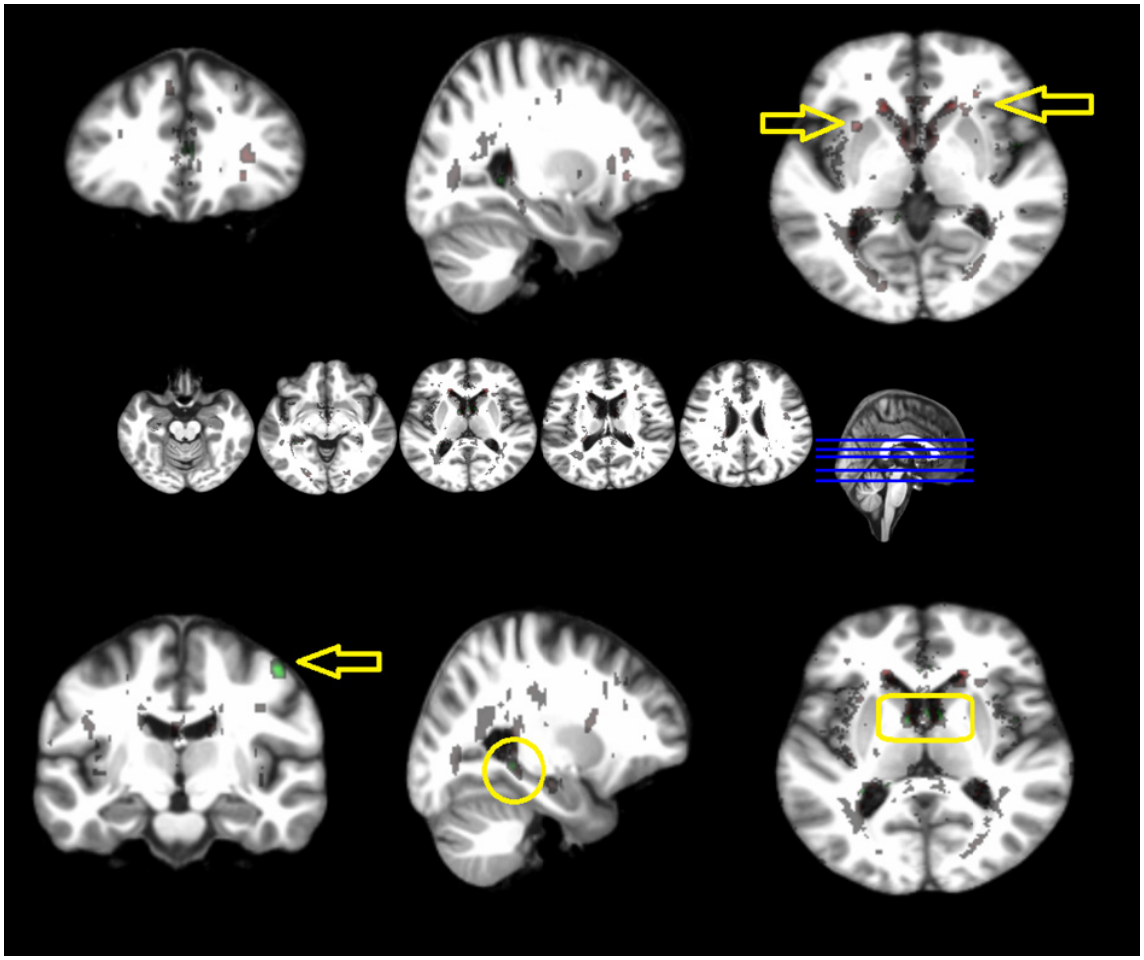

Supplementary Figure 10 Illustration of the voxel-based association between WMH and fibrinolysis (D-dimer) showing the brain voxels of the study template where positive (green) and negative (red) associations were found in representative slices in the three orientation planes. The colour intensity is proportional to the strength of the association (normalised to values between 0-256 for visual representation). Arrowed and encircled, in the bottom row, are the clusters where WMH were most strongly associated with this blood marker.

References

Andersson JLR, Jenkinson M, Smith S. Non-linear registration aka spatial normalisation. FMRIB Tech Rep TR07JA2. 2007;(June). Available at: http://fmrib.medsci.ox.ac.uk/analysis/techrep/tr07ja2/tr07ja2.pdf.

Behrens TEJ, Berg HJ, Jbabdi S, Rushworth MFS, Woolrich MW. Probabilistic diffusion tractography with multiple fibre orientations: What can we gain? NeuroImage 2007; 34:144–155. doi:10.1016/j.neuroimage.2006.09.018.

Behrens TEJ, Woolrich MW, Jenkinson M, Johansen-Berg H, Nunes RG, Clare S, Matthews PM, Brady JM, and Smith SM. Characterization and propagation of uncertainty in diffusion-weighted MR imaging. Magn Reson Med 2003; 50(5):1077-1088.

Bullitt E, Gerig G, Pizer SM, Lin W, Aylward, SR. Measuring tortuosity of the intracerebral vasculature from MRA images. IEEE Trans Med Imaging 2008;22:1163–71. doi:10.1109/TMI.2003.816964.

Dormann CF, Fründ J, Bluthgen N, Grüber B. Indices, graphs and null models: Analyzing bipartite ecological networks. Open Ecology J 2009; 2:7–24.

Jenkinson M, Smith S. A global optimization method for robust affine registration of brain images. Med Image Anal 2001; 5:143–56.

Modat M, Ridgway GR, Taylor ZA, Lehmann M, Barnes J, Hawkes DJ, et al. Fast free-form deformation using graphics processing units. Comput Methods Programs Biomed 2010;8:278–84. doi:10.1016/j.cmpb.2009.09.002.

Smith K, Abasolo D, Escudero J. Accounting for the complex hierarchical topology of EEG phase-based functional connectivity in network binarisation. PLOS One 2017;12:e0186164. https://doi.org/10.1371/journal.pone.0186164.

Smith K, Bastin ME, Cox SR, Valdés Hernández MC, Wiseman SJ, Escudero J, et al. Hierarchical complexity of the adult human structural connectome. NeuroImage 2019;191:205–215. https://doi.org/10.1016/j.neuroimage.2019.02.028
